# Height, body mass index, and socioeconomic status: mendelian randomisation study in UK Biobank

**DOI:** 10.1136/bmj.i582

**Published:** 2016-03-08

**Authors:** Jessica Tyrrell, Samuel E Jones, Robin Beaumont, Christina M Astley, Rebecca Lovell, Hanieh Yaghootkar, Marcus Tuke, Katherine S Ruth, Rachel M Freathy, Joel N Hirschhorn, Andrew R Wood, Anna Murray, Michael N Weedon, Timothy M Frayling

**Affiliations:** 1 Genetics of Complex Traits, Institute of Biomedical and Clinical Science, University of Exeter Medical School, Royal Devon and Exeter Hospital, Exeter EX2 5DW, UK; 2 European Centre for Environment and Human Health, University of Exeter Medical School, The Knowledge Spa, Truro TR1 3HD, UK; 3 Program in Medical and Population Genetics, Broad Institute of MIT and Harvard, Cambridge, MA 02142, USA; 4 Center for Basic and Translational Obesity Research and Division of Endocrinology, Boston Children’s Hospital, Boston, MA 02115, USA; 5 Department of Genetics, Harvard Medical School, Boston, MA 02115, USA

## Abstract

**Objective** To determine whether height and body mass index (BMI) have a causal role in five measures of socioeconomic status.

**Design** Mendelian randomisation study to test for causal effects of differences in stature and BMI on five measures of socioeconomic status. Mendelian randomisation exploits the fact that genotypes are randomly assigned at conception and thus not confounded by non-genetic factors.

**Setting** UK Biobank.

**Participants** 119 669 men and women of British ancestry, aged between 37 and 73 years.

**Main outcome measures** Age completed full time education, degree level education, job class, annual household income, and Townsend deprivation index.

**Results** In the UK Biobank study, shorter stature and higher BMI were observationally associated with several measures of lower socioeconomic status. The associations between shorter stature and lower socioeconomic status tended to be stronger in men, and the associations between higher BMI and lower socioeconomic status tended to be stronger in women. For example, a 1 standard deviation (SD) higher BMI was associated with a £210 (€276; $300; 95% confidence interval £84 to £420; P=6×10^−3^) lower annual household income in men and a £1890 (£1680 to £2100; P=6×10^−15^) lower annual household income in women. Genetic analysis provided evidence that these associations were partly causal. A genetically determined 1 SD (6.3 cm) taller stature caused a 0.06 (0.02 to 0.09) year older age of completing full time education (P=0.01), a 1.12 (1.07 to 1.18) times higher odds of working in a skilled profession (P=6×10^−7^), and a £1130 (£680 to £1580) higher annual household income (P=4×10^−8^). Associations were stronger in men. A genetically determined 1 SD higher BMI (4.6 kg/m^2^) caused a £2940 (£1680 to £4200; P=1×10^−5^) lower annual household income and a 0.10 (0.04 to 0.16) SD (P=0.001) higher level of deprivation in women only.

**Conclusions** These data support evidence that height and BMI play an important partial role in determining several aspects of a person’s socioeconomic status, especially women’s BMI for income and deprivation and men’s height for education, income, and job class. These findings have important social and health implications, supporting evidence that overweight people, especially women, are at a disadvantage and that taller people, especially men, are at an advantage.

## Introduction

Higher socioeconomic status is associated with better health and longer life.^1^ ^2^ For example, a recent article highlighted the strength of the association between wealth and health by pointing out the 18 and 20 year gaps in male life expectancy between the least and most wealthy parts of London, UK, and Baltimore, USA, respectively.^3^ Two easily measured markers associated with socioeconomic status are adult height and body mass index ( BMI).^4^ ^5^ ^6^ In developed counties, taller stature and lower BMI are associated with higher socioeconomic status and better health.^4^ ^5^ ^6^ ^7^ ^8^ ^9^ ^10^ ^11^ ^12^ Higher socioeconomic status is generally thought to cause taller stature and lower BMI owing to higher standards of nutrition in childhood, but there may also be effects in the opposite direction—taller stature and lower BMI may causally improve socioeconomic status through discrimination against shorter and fatter people or differences in self esteem that affect employability.^13^ ^14^ Evidence as to whether height and BMI have causal effects on socioeconomic status through these, or other, pathways is limited. For example, to our knowledge, no large studies have compared siblings or twins of different heights and BMIs, where childhood environment could be controlled for. If differences in BMI and height can lead to differences in socioeconomic status, this would have implications for policy makers. For example, evidence of a causal link would further highlight the need to adjust for unconscious biases in decision making in education and employment.

Gene based analyses, such as mendelian randomisation,^15^ can be used to test for a causal relation between socioeconomic status and a genetically influenced phenotype such as BMI. Genetic variants can act as unconfounded proxies for the risk factors under investigation—here, BMI and height—because inherited genetic variation is randomly allocated at conception. The outcomes being tested—here, measures of socioeconomic status—cannot influence genetic variation, so reverse causality is avoided in genetic studies. Figure 1 illustrates the principle of mendelian randomisation. Previous studies have used genetic variants to test causal relation between health traits such as BMI and socioeconomic status related outcomes such as academic performance. However, these studies were limited by a lack of genetic variants robustly associated with BMI and by sample sizes of fewer than 2300 people.^16^ ^17^ Recent genome-wide association studies have identified many 10s and 100s of genetic variants associated with BMI and height, respectively,^18^ ^19^ and so provide the tools for mendelian randomisation tests.

**Figure f1:**
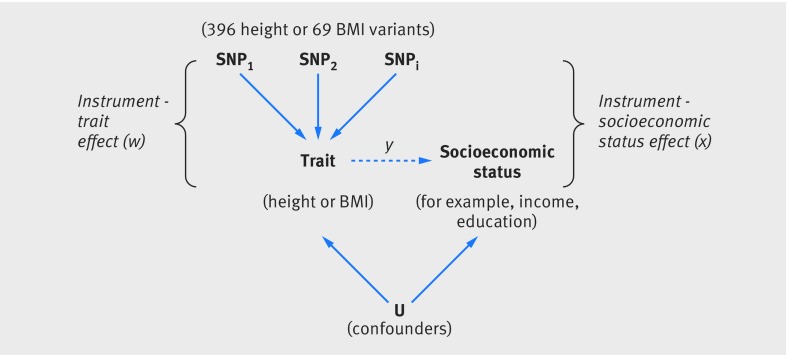
**Fig 1** Principle of mendelian randomisation: if height or body mass index (BMI) causally influences socioeconomic status, genetic variants associated with that trait will also be associated with socioeconomic status. As genotype is assigned at conception, it should not be associated with factors that normally confound the association between BMI and height and socioeconomic status (eg, environmental and behavioural factors). We can use our estimates of the genetic-height/BMI association (w) and the genetic-socioeconomic status association (x) to infer the causal effect of height or BMI on socioeconomic status (y=x/w), which is expected to be free from confounding. If the estimated causal effect (y) is different from the observational association between the height or BMI and socioeconomic status, this would suggest that the observational association is confounded (assuming that the assumptions of the mendelian randomisation analyses are valid). SNP=single nucleotide polymorphism

Here, we used mendelian randomisation analysis to test the hypothesis that causal pathways link BMI and height to differences in five different measures of socioeconomic status. We used the first release of data from the UK Biobank. The UK Biobank has 119 669 participants of white British ancestry with genetic data, measures of socioeconomic status, and height and BMI measures. The UK Biobank thus represents a very powerful resource in which to investigate the causal relation between BMI, height, and socioeconomic status by using mendelian randomisation analysis.

## Methods

### UK Biobank

The UK Biobank recruited more than 500 000 people aged 37-73 years (99.5% were between 40 and 69 years) from across the country in 2006-10. Participants provided a range of information via questionnaires and interviews (such as demographics, health status, and lifestyle); anthropometric measurements, blood pressure readings, and blood, urine and saliva samples were taken for future analysis. This has been described in more detail elsewhere.^20^ We used 120 286 participants of white British descent from the initial UK Biobank dataset, of whom 119 669 had valid genetic data and both BMI and height measures available. We did not include other ethnic groups, because individually they were underpowered. Table 1 shows the basic characteristics of the sample. We defined people of white British descent as those who both self identified as white British and were confirmed as ancestrally “Caucasian” using principal components analyses of genome-wide genetic information. This dataset underwent extensive central quality control (http://biobank.ctsu.ox.ac.uk) (see supplementary methods).

**Table 1 tbl1:** Summary of demographic characteristics of 119 669 participants of white British ancestry with valid genetic data and height and body mass index measures available, stratified by sex. Values are numbers (percentages) unless stated otherwise

Characteristic	All (n=119 669)	Men (n=56 652)	Women (n=63 017)	P value*
Mean (SD) age at recruitment, years	56.9 (7.9)	57.3 (8.0)	56.6 (7.8)	<1×10^−15^
Male sex	56 652 (47.3)	56 652 (100)	63 017 (100)	–
Mean (SD) body mass index, kg/m^2^	27.5 (4.8)	27.9 (4.3)	27.2 (5.2)	<1×10^−15^
Mean (SD) height, cm	168.8 (9.2)	175.7 (6.7)	162.6 (6.2)	<1×10^−15^
Smoking status:				
Never	63 806 (53.3)	27 834 (49.1)	35 972 (57.1)	<1×10^−15^
Former	40 890 (34.2)	21 162 (37.4)	19 728 (31.3)	
Current	13 332 (11.1)	6767 (11.9)	6565 (10.4)	
Missing	1641 (1.4)	889 (1.6)	752 (1.2)	
Mean (SD) age completed full time education, years	16.6 (2.2)	16.6 (2.4)	16.5 (2.0)	2×10^−9^
Degree level education	53 652 (44.8)	25 956 (45.8)	27 696 (44.0)	6×10^−15^
Job class:				
Elementary occupations	3932 (3.3)	2054 (3.6)	1878 (3.0)	<1×10^−15^
Process plant and machine operatives	3740 (3.1)	3299 (5.8)	441 (0.7)	
Sales and customer service occupations	2658 (2.2)	588 (1.0)	2070 (3.3)	
Leisure and other personal service occupations	963 (0.8)	379 (0.7)	584 (0.9)	
Personal service occupations	3567 (3.0)	404 (0.7)	3163 (5.0)	
Skilled trades	6077 (5.1)	5404 (9.5)	673 (1.1)	
Administrative and secretarial roles	11 878 (9.9)	2329 (4.1)	9549 (15.2)	
Business and public sector associate professionals	4631 (3.9)	2548 (4.5)	2083 (3.3)	
Associate professionals	8388 (7.0)	3148 (5.6)	5240 (8.3)	
Professional occupations	17 044 (14.2)	8934 (15.8)	8110 (12.9)	
Senior officials	13 526 (11.3)	8521 (15.0)	5005 (7.9)	
Income:				
<£18 000	23 817 (19.9)	10 499 (18.5)	13 318 (21.1)	<1×10^−15^
£18 000 to £30 999	26 808 (22.4)	12 788 (22.6)	14 020 (22.3)	
£31 000 to £51 999	27 245 (22.8)	13 848 (24.4)	13 397 (21.3)	
£52 000 to £100 000	20 397 (17.0)	10 950 (19.3)	9447 (15.0)	
>£100 000	5060 (4.2)	2777 (4.9)	2283 (3.6)	
Mean (SD) Townsend deprivation index	−1.5 (3.0)	−1.51 (3.0)	−1.45 (2.9)	<1×10^−15^
Overall per allele height SNP association with height	0.021 (0.021 to 0.022); P<1×10^−15^	0.022 (0.022 to 0.023); P<1×10^−15^	0.020 (0.020 to 0.021); P<1×10^−15^	–
Overall per allele BMI SNP association with BMI	0.022 (0.021 to 0.023); P<1×10^−15^	0.022 (0.021 to 0.024); P<1×10^−15^	0.025 (0.023 to 0.026); P<1×10^−15^	–

BMI=body mass index; SNP=single nucleotide polymorphism.

Not all five socioeconomic status measures were available in all 119 669 individuals (see supplementary table A for further information).

*For comparison between men and women; models were adjusted for age at recruitment.

### Patient involvement

This study was conducted using the UK Biobank resource. Details of patient and public involvement in the UK Biobank are available online (www.ukbiobank.ac.uk/about-biobank-uk/ and https://www.ukbiobank.ac.uk/wp-content/uploads/2011/07/Summary-EGF-consultation.pdf?phpMyAdmin=trmKQlYdjjnQIgJ%2CfAzikMhEnx6). No patients were specifically involved in setting the research question or the outcome measures, nor were they involved in developing plans for recruitment, design, or implementation of this study. No patients were asked to advise on interpretation or writing up of results. There are no specific plans to disseminate the results of the research to study participants, but the UK Biobank disseminates key findings from projects on its website.

### Exposure and outcome measures

Exposure and outcome measures were all collected at baseline when participants attended the assessment centre.

#### Height

Height (cm) was measured using a Seca 202 device in all participants in the UK Biobank (n=500 120). Sitting height was also measured (n=496 380). We excluded one person with a height more than 4.56 SD away from the mean and a sitting height to standing height ratio of greater than 0.75, which is not compatible with normal growth. In all, 119 669 people of white British ancestry with genetic data available also had a valid height and BMI measure.

#### Body mass index

The UK Biobank has two different measures of BMI—one calculated as weight/height^2^ and one measured using electrical impedance to quantify mass. We excluded people with significant differences (>4.56 SD from the mean) between impedance and normal BMI measures (n=1172) where both variables were available. If only one measure of BMI was available, we used this (n=7290). Valid BMI was available for 119 669 people with genetic and height data available.

#### Socioeconomic status

We used five different socioeconomic status variables.^1^ Age in years at completion of full time education (questionnaire based; available only for people who did not go on to degree level education). Data were available for 82 543 people and missing in 37 126 people with valid height, BMI, and genetic data.^2^ Education (coded as degree level or not, derived from the questionnaire); participants were asked, “Which of the following qualifications do you have? (You can select more than one),” with the options college or university degree, A levels or equivalent, O levels or GCSEs or equivalent, CSEs, NVQ/HND/HNC, professional qualifications (eg, nursing or teaching). We created a dichotomous variable comparing degree level education or professional qualifications (n=53 652) with other qualifications (n=64 913); 1104 people did not respond to this question.^3^ Job class (coded as elementary occupations, process plant and machine operatives, sales and customer service occupations, leisure and other personal service occupations, personal service occupations, skilled trades, administrative and secretarial roles, business and public sector associate professionals, associate professionals, professional occupations, and managers and senior officials); this was coded from the UK Biobank job code variable. All participants were asked to select their current or most recent job. Data were available for 76 404 people, with missing data in 43 265. We dichotomised this variable into unskilled (n=21 036; elementary occupations to personal service occupations) and skilled (n=55 698; skilled trades to managers and senior officials).^4^ A categorical income variable (questionnaire based), representing annual household income of <£18 000 (€23 600; $25 800), £18 000 to £30 999, £31 000 to £51 999, £52 000 to £100 000, and >£100 000. Data were available for 103 327 people and missing in 16 432 people with valid height, BMI, and genetic data.^5^ Townsend deprivation index (a composite measure of deprivation based on unemployment, non-car ownership, non-home ownership, and household overcrowding; a negative value represents high socioeconomic status). This was calculated before participants joined the UK Biobank and was based on the preceding national census data, with each participant assigned a score corresponding to the postcode of their home dwelling. Data were available for 119 519 people and missing in 150 people with valid height, BMI, and genetic data.

For each of the five traits, we compared participants missing data with those reporting data; generally, those with missing data were older and shorter with higher BMIs (supplementary table A). We investigated the relation of these five socioeconomic status measures and four health outcomes: self reported coronary artery disease, hypertension (defined as a systolic blood pressure ≥140, a diastolic blood pressure ≥90, or the report of use of blood pressure lowering drugs), any self reported long term illness (based on the UK Biobank question “Do you have a long standing illness, disability or infirmity?”), and type 2 diabetes (based on self report, excluding people using insulin in the first year after diagnosis and those given a diagnosis before 35 years of age or within the previous year) (supplementary table B).

For three of the traits (the exceptions being education and job class, both binary traits), we converted the data to a normal distribution to limit the influence of any subtle population stratification and to provide standard deviation effect sizes. We took residuals of the exposure and outcome measures from standard linear regression by using nine covariates: age, sex, assessment centre location, five (within UK) ancestry principal components, and microarray used to measure genotypes. We then inverse normalised the residualised variables. To convert our results back to meaningful units after mendelian randomisation, we multiplied our SD βs by a 1 SD change in the socioeconomic status measure. For example, a 1 SD change in Townsend deprivation index was equivalent to 2.68 units. Therefore, a 0.05 SD change equated to a 0.134 unit change in deprivation.

### Observational associations

We regressed each socioeconomic status measure against height and BMI by using linear regression for continuous outcome variables and logistic regression for binary outcomes. We adjusted these associations for age and sex. We also investigated the association of each socioeconomic status measure with a range of health outcomes.

### Genetic variants

The genetic variants used were extracted genotypes from UK Biobank’s imputation dataset (the supplementary methods provide more information on the UK Biobank’s quality control). We excluded individual genotypes if the genotype probability was less than 0.9. We confirmed that the variants were imputed with high quality by comparing them with the directly genotyped data, where available. Details of imputation quality are given in supplementary table C.

*Height—*We selected 396 of 404 height genetic variants from independent loci that were associated with height at genome-wide significance in the GIANT studies of up to 253 288 people (supplementary table C).^19^ We excluded eight variants that were unavailable (rs1420023, rs567401), were poorly imputed, had an imputation quality <0.9 (rs11683207, rs7534365), or were not in Hardy-Weinberg equilibrium (P<1×10^−6^; rs1401795, rs7692995, rs915506, rs3790086). The 396 variants explained 12.3% of the variance in adult height in the UK Biobank participants used.

*Body mass index—*We selected 69 of 76 common genetic variants that were associated with BMI at genome-wide significance in the GIANT consortium in studies of up to 339 224 people (supplementary table C).^18^ We limited the BMI variants to those that were associated with BMI in the analysis of all people of European ancestry and did not include those that reached genome-wide levels of statistical confidence in only one sex or one stratum. We also excluded variants if they were known to be classified as a secondary signal within a locus. Three variants were excluded from the score owing to potential pleiotropy (rs11030104 (*BDNF* reward phenotypes), rs13107325 (*SLC39A8* lipids, blood pressure), rs3888190 (*SH2B1* multiple traits)), three were not in Hardy-Weinberg equilibrium (P<1×10^−6^ ; rs17001654, rs2075650, rs9925964), and one was unavailable (rs2033529). The 69 variants explained 1.5% of the variance in BMI in the UK Biobank participants.

We recoded individual variants as 0, 1, and 2 according to the number of height or BMI increasing alleles. We used the variants to create height and BMI genetic risk scores. Each variant was weighted by its relative effect size (β coefficient) obtained from the reported meta-analysis data.^18^ We created a weighted score, in which β is the β coefficient of representing the association between each single nucleotide polymorphism (SNP) and height/BMI: weighted score=β_1_×SNP_1_+β_2_×SNP_2_+⋯β_n_×SNP_n_. We then rescaled the weighted score to reflect the number of trait increasing alleles: weighted genetic risk score=(weighted score×number of SNPs)/sum of β coefficients.

### Mendelian randomisation

The mendelian randomisation approach used in this study made the following assumptions^15^: the height and BMI genetic risk scores were robustly associated with measured height and BMI; the height and BMI genetic risk scores were not associated with confounding factors that bias conventional epidemiological associations between height/BMI and socioeconomic status; the height and BMI genetic risk scores were related to the outcome only via its association with the modifiable exposure; and the associations represented in figure 1 are linear and unaffected by statistical interactions.

#### Instrumental variable analysis

We used two methods that use genetic variants to assess causal relations between two traits. Firstly, to estimate the causal effect of height or BMI on individual socioeconomic status measures, we used instrumental variable analysis using the height or BMI genetic risk score.^15^ We used the two stage, least squares estimator method that uses predicted levels of BMI or height per genotype and regresses each outcome against these predicted values.

For continuous socioeconomic status outcomes, we used the ivreg2 command in Stata to do the instrumental variable analysis. We compared results from observational and instrumental variable regressions by using the Durbin-Wu-Hausman test for endogeneity, which examines the difference between the estimates from linear regression (observational) and instrumental variable analysis.^21^

For binary outcomes, the instrumental variable analysis was done in two stages. Firstly, we assessed the association between the height or BMI genetic risk score and height or BMI, respectively. We saved the predicted values and residuals from this regression model. Secondly, we used the predicted values from stage 1 as the independent variable (reflecting an unconfounded estimate of variation in BMI or height) and degree status or job class as the dependent variable in a logistic or ordinal logistic regression model. We used robust standard errors to correct for uncertainty in the estimate. We examined the F statistics from first stage regressions to evaluate the strength of the instruments; weak instruments can bias results towards the (confounded) multivariable regression association or towards the null in a two stage design.^22^ ^23^

#### Egger method

We used a second method of mendelian randomisation, the Egger method,^24^ as a sensitivity analysis if the instrumental variables test result was noteworthy. This method is more robust to potential violations of the standard instrumental variable assumptions. It uses a weighted regression with an unconstrained intercept to regress the effect sizes of variant-outcome associations (here, height or BMI variants versus socioeconomic status measures) against effect sizes of variant-risk factor associations (here, height or BMI variants versus height or BMI). The unconstrained intercept removes the assumption that all genetic variants are valid instrumental variables, so this method is less susceptible to confounding from potentially pleiotropic variants that will probably have stronger effects on outcomes compared with their effects on the primary trait. The approach is analogous to correcting for small study publication bias in meta-analyses.^24^ Details of the Stata and R code used are provided in Bowden et al 2015.^24^

To ensure the robustness of our findings, we have highlighted results only where we see consistent results across the two different methods.

### Differences between men and women

To test the hypothesis that the effects of height and BMI on socioeconomic status may differ in men and women, we repeated observational and genetic analyses separately in each sex. The selected height and BMI genetic variants have very similar effects in men and women, so we used the same genetic risk scores in all participants, men only, and women only. We compared the β values for men and women by using Fisher’s z score method^25^: z=(β_1_−β_2_)/√(SE1^2^+SE2^2^).

### Sensitivity analyses

In a sensitivity analysis to further confirm that our results were robust to any potential influence of population stratification, we used the linear mixed models approach as implemented in the software BOLT-LMM.^26^ This approach corrects for all levels of inter-individual correlation of genotypes due to relatedness, from close relatives to cryptic relatedness caused by population stratification. We inverse normalised the socioeconomic status measures, then took the residuals using three covariates (age, sex, assessment centre location) and inverse normalised again.

## Results

Table 1 summarises the demographics of the 119 669 UK Biobank participants with valid genetic data and BMI and height measures. The height and BMI genetic risk scores were robustly associated with height and BMI (table 1). The association between the socioeconomic status measures and health outcomes and the associations between known height variants and height and known BMI variants and BMI in the UK Biobank are summarised in supplementary tables A-C.

### Relation of genetically determined taller stature to higher socioeconomic status measures in UK Biobank

#### Education: duration in full time education

Using 82 543 participants, we found that taller stature was strongly correlated with participants spending longer in full time education (table 2). This association was similar in men and women. A 1 SD (6.3 cm) greater height was associated with a 0.11 (95% confidence interval 0.10 to 0.12) SD older age (approximately 0.2 years) at which full time education was completed. Genetic analyses provided evidence that this association was partly causal—a genetically determined 1 SD (6.3 cm) higher height was associated with a 0.03 (0.01 to 0.05) SD older age (approximately 0.06 (0.02 to 0.10) years) at which full time education was completed (table 2, supplementary figure A-I).

**Table 2 tbl2:** Associations between taller stature and five measures of socioeconomic, using linear or logistic regression and instrumental variable analysis

Socioeconomic status measures and subcategories	No	Observational*			Genetic†			Genetic: Egger‡	
		Change in socioeconomic status (95%CI) per SD taller stature	P value		Change in socioeconomic status (95%CI) per SD taller stature	P value		Change in socioeconomic status (95%CI) per SD taller stature	P value
Age completed full time education:									
All	82 543	0.11 (0.10 to 0.12)	<1×10^−15^		0.03 (0.01 to 0.05)	0.01		0.07 (0.03 to 0.11)	0.0004
Men only	38 342	0.11 (0.10 to 0.12)	<1×10^−15^		0.04 (0.01 to 0.07)	0.009		0.08 (0.02 to 0.14)	0.004
Women only	44 201	0.11 (0.10 to 0.12)	<1×10^−15^		0.01 (−0.02 to 0.04)	0.40		–	
Degree level education:									
All	118 565	OR: 1.25 (1.24 to 1.27)	<1×10^−15^		1.02 (0.99 to 1.05)	0.22		–	
Men only	56 111	OR: 1.25 (1.23 to 1.27)	<1×10^−15^		1.04 (1.00 to 1.09)	0.08		–	
Women only	62 454	OR: 1.26 (1.24 to 1.28)	<1×10^−15^		1.00 (0.95 to 1.05)	0.97		–	
Job class (skilled/unskilled):									
All	76 404	OR: 1.29 (1.27 to 1.32)	<1×10^−15^		1.12 (1.07 to 1.18)	6E−7		1.18 (1.08 to 1.29)	0.0002
Men only	37 608	OR: 1.31 (1.28 to 1.34)	<1×10^−15^		1.13 (1.07 to 1.21)	2E−5		1.23 (1.10 to 1.37)	0.0004
Women only	38 796	OR: 1.27 (1.24 to 1.31)	<1×10^−15^		1.14 (1.05 to 1.24)	0.003		1.21 (1.08 to 1.36)	0.002
Annual household income:									
All	103 327	0.13 (0.12 to 0.14)	<1×10^−15^		0.05 (0.03 to 0.07)	4E−8		0.05 (0.02 to 0.08)	0.0009
Men only	50 862	0.15 (0.14 to 0.16)	<1×10^−15^		0.07 (0.05 to 0.10)	1E−9		0.08 (0.04 to 0.12)	0.0002
Women only	52 465	0.11 (0.10 to 0.12)	<1×10^−15^		0.02 (0.00 to 0.05)	0.09		–	
Townsend deprivation index:									
All	119 519	−0.08 (−0.09 to −0.07)	<1×10^−15^		0.00 (−0.02 to 0.01)	0.71		–	
Men only	56 582	−0.10 (−0.10 to −0.09)	<1×10^−15^		−0.02 (−0.05 to 0.00)	0.05		−0.08 (−0.12 to −0.04)	0.0004
Women only	62 937	−0.07 (−0.07 to −0.06)	<1×10^−15^		0.02 (−0.01 to 0.04)	0.19		–	

OR=odds ratio.

For age completed full time education, annual household income, and Townsend deprivation index, changes reported are standard deviation. For degree and job class, odds ratios are shown, representing odds of higher socioeconomic status per SD greater height.

*Age, assessment centre, and sex adjusted associations.

†Uses instrumental variable analysis via ivreg2 command in Stata for continuous variables and two step procedure for binary outcomes using height genetic risk score. F statistic when considering all participants is ≥10 898 for each socioeconomic status measure; in men only, F statistic is ≥5308 for each socioeconomic status measure; in women only, F statistic is ≥5615 for each socioeconomic status measure.

‡Alternative genetic approach detailed in Bowden et al 2015,^24^ used as sensitivity analysis when instrumental variable was P<0.05.

#### Education: degree level (or equivalent) or not

Using 118 565 participants, we found that taller stature was strongly correlated with participants’ chances of having obtained a degree. A 1 SD (6.3 cm) greater height was associated with 1.25 (1.24 to 1.27) increased odds of reporting degree level education. This association was similar in men and women (P for comparison>0.05). Genetic analyses provided no consistent evidence for a causal role of height in obtaining degree level education (table 2, supplementary figure A-II).

#### Job class

Using 76 404 participants, we found that taller stature was strongly correlated with job class. A 1 SD (6.3 cm) greater height was associated with increased odds of working in skilled job roles (odds ratio 1.29, 1.27 to 1.32). Genetic analyses provided evidence that this association was partly causal—a 1 SD (6.3 cm) genetically determined higher height was associated with increased odds of working in more professional roles (odds ratio 1.12, 1.07 to 1.18) (table 2, supplementary figure A-III). This association was consistent when we analysed the data as 11 ordered job classes (supplementary table D). We found no genetic evidence that the effect was stronger in men or women.

#### Annual household income

Using 103 327 participants, we found that taller stature was strongly correlated with higher household income. The correlation was approximately 50% stronger in men (table 2). A 1 SD (6.3 cm) greater height was associated with a 0.13 (0.12 to 0.14) SD increase in income (table 2). This difference is approximately equivalent to a £2940 (£2730 to £3185) higher annual household income. Genetic analyses provided evidence that this association was partly causal—a genetically determined 1 SD (6.3 cm) greater height was associated with a 0.05 (0.03 to 0.07) SD increase in annual household income, equivalent to £1130 (£680 to £1580) (table 2 and supplementary figure AIV). The genetic analyses showed that the effect was approximately twice as strong in men as in women (P for comparison=5×10^−4^); a 1 SD greater height in men caused a £1580 (£1130 to £2260) increase in household income (supplementary figure A-IV). This association was consistent when we analysed the data as five ordered income classes (supplementary table D).

#### Townsend deprivation index

Using 119 519 participants, we found that taller stature was strongly correlated with lower levels of social deprivation, as measured by the Townsend deprivation index. This association was stronger in men than women. A 1 SD (6.3 cm) greater height was associated with a 0.08 (0.07 to 0.09) SD lower deprivation, which is equivalent to a 0.21 (0.18 to 0.24) unit reduction in Townsend deprivation index (table 2). Genetic analyses provided evidence that this association was partly causal in men but not when all participants or women were considered. In all participants, genetically determined height was not associated with deprivation (table 2, supplementary figure A-V). In men, a genetically determined 1 SD (6.3 cm) greater height was associated with a 0.02 (0.00 to 0.05) SD reduction in deprivation (supplementary figure AV). This difference is equivalent to a 0.05 (0.00 to 0.13) unit lower Townsend index.

### Relation of genetically determined higher BMI to reduced income and deprivation measures of socioeconomic status in UK Biobank

#### Education: duration in full time education

Using 82 543 participants, we found that higher BMI was strongly correlated with participants finishing full time education at a younger age. The association was similar in men and women (P for comparison>0.05) (table 3). A 1 SD (4.6 kg/m^2^) higher BMI was associated with a 0.08 (0.07 to 0.08) SD younger age (approximately 0.15 years) at which full time education was completed. We found no genetic evidence that this association was causal when considering all participants, men only, or women only (supplementary figure B-I).

**Table 3 tbl3:** Associations between higher BMI and five measures of socioeconomic, using linear or logistic regression and instrumental variable analysis

Socioeconomic status measures and subcategories	No	Observational*			Genetic†			Genetic: Egger‡	
		Change in socioeconomic status (95%CI) per SD higher BMI	P value		Change in socioeconomic status (95%CI) per SD higher BMI	P value		Change in socioeconomic status (95%CI) per SD higher BMI	P value
Age completed full time education:									
All	82 543	−0.08 (−0.08 to −0.07)	<1×10^−15^		−0.01 (−0.07 to 0.04)	0.63		–	
Men only	38 342	−0.07 (−0.08 to −0.06)	<1×10^−15^		0.00 (−0.09 to 0.09)	0.98		–	
Women only	44 201	−0.08 (−0.09 to −0.07)	<1×10^−15^		−0.02 (−0.09 to 0.05)	0.56		–	
Degree level education:									
All	118 565	OR: 0.83 (0.82 to 0.84)	<1×10^−15^		0.94 (0.85 to 1.03)	0.18		–	
Men only	56 111	OR: 0.82 (0.81 to 0.84)	<1×10^−15^		0.94 (0.81 to 1.09)	0.43		–	
Women only	62 454	OR: 0.83 (0.82 to 0.84)	<1×10^−15^		0.93 (0.82 to 1.06)	0.28		–	
Job class (skilled/unskilled):									
All	76 404	OR: 0.91 (0.89 to 0.92)	<1×10^−15^		0.90 (0.79 to 1.02)	0.10		–	
Men only	37 608	OR: 0.93 (0.91 to 0.95)	8×10^−9^		0.88 (0.73 to 1.08)	0.22		–	
Women only	38 796	OR: 0.89 (0.87 to 0.91)	<1×10^−15^		0.91 (0.76 to 1.08)	0.29		–	
Annual household income:									
All	103 327	−0.06 (−0.06 to −0.05)	<1×10^−15^		−0.05 (−0.10 to −0.00)	0.041		−0.03 (−0.11 to 0.05)	0.58
Men only	50 862	−0.01 (−0.02 to −0.00)	<1×10^−15^		0.06 (−0.02 to 0.14)	0.15		–	
Women only	52 465	−0.09 (−0.10 to −0.08)	<1×10^−15^		−0.14 (−0.20 to −0.08)	1×10^−5^		−0.17 (−0.25 to −0.05)	0.004
Townsend deprivation index:									
All	119 519	0.08 (0.07 to 0.08)	<1×10^−15^		0.05 (0.01 to 0.10)	0.024		−0.00 (−0.08 to 0.08)	0.96
Men only	56 582	0.05 (0.04 to 0.05)	<1×10^−15^		−0.01 (−0.08 to 0.06)	0.78		–	
Women only	62 937	0.10 (0.09 to 0.11)	<1×10^−15^		0.10 (0.04 to 0.16)	0.001		0.10 (−0.01 to 0.21)	0.08

BMI=body mass index; OR=odds ratio.

For age completed full time education, annual household income, and Townsend deprivation index, changes reported are standard deviation. For degree and job class, odds ratios are shown, representing odds of higher socioeconomic status per SD higher BMI.

*Age, assessment centre, and sex adjusted associations.

†Uses instrumental variable analysis, via ivreg2 command in Stata for continuous variables and two step approach for binary outcomes, using BMI genetic risk score. F statistic for all participants is ≥1257 for each socioeconomic status measure; in men only, F statistic is ≥591 for each socioeconomic status measure; in women only, F statistic is ≥666 for each socioeconomic status measure.

‡Alternative genetic approach detailed in Bowden et al 2015,^24^ used as sensitivity analysis when instrumental variable was P<0.05.

#### Education: degree level (or equivalent or not)

Using 118 565 participants, we found that higher BMI was associated with lower odds of having obtained a degree. A 1 SD higher BMI was associated with lower odds of obtaining degree level education (odds ratio 0.83, 0.82 to 0.84). We found no consistent genetic evidence that this association was causal when considering all participants, men only, or women only (supplementary figure B-II).

#### Job class

Using 76 404 participants, we found that higher BMI was associated with employment in less skilled professions. A 1 SD (4.6 kg/m^2^) higher BMI was associated with lower odds of working in skilled job roles (0.91, 0.89 to 0.92), and the association was stronger in women. We found no consistent genetic evidence that this association was causal when considering all participants, men only, or women only (supplementary figure B-III). However, we found some evidence of causality when we analysed the data as 11 ordered job classes (supplementary table D).

#### Annual household income

Using 103 327 participants, we found that higher BMI was associated with a lower annual household income, but this effect was very strongly driven by the association in women. A 1 SD higher BMI was associated with a 0.09 (0.08 to 0.10) SD lower household income for women. This effect equates to £1890 (£1680 to £2100) less income per annum for women. In men, a 1 SD higher BMI approximated to a £210 (£84 to £420) lower annual household income. Genetic analyses were consistent with these observations being causal in women but not in men (P for comparison with men=9×10^−5^)—a genetically determined 1 SD higher BMI was associated with an annual household income of 0.14 (0.08 to 0.20) SD less in women. This effect is equivalent to £2940 (£1680 to £4200) less for women (table 3, supplementary figure B-IV). This association was consistent when we analysed the data as five ordered income classes (supplementary table D). The association between higher BMI and lower income was consistent in women who worked, with or without a husband/partner at home, and women who did not work with a husband/partner at home (supplementary table E). It was also consistent when we considered only women without health conditions (supplementary table E).

#### Townsend deprivation index

Higher BMI was associated with higher levels of deprivation as assessed by the Townsend deprivation index. A 1 SD higher BMI was associated with a 0.08 (0.07 to 0.08) SD higher deprivation value, which is equivalent to a 0.21 (0.19 to 0.21) unit increase in Townsend index (table 3). This association was twice as strong in women. We found limited genetic evidence of a causal relation between BMI and deprivation in men, but some evidence in women. A 1 SD genetically higher BMI was associated with a 0.10 (0.04 to 0.16) SD higher level of deprivation in women (table 3, Supplementary figure B-V).

### Sensitivity analyses

The Egger method provided consistent results for causal relations between height and duration in full time education, job class, income, and Townsend deprivation index in men (table 2, supplementary table F). The Egger method also provided evidence of consistent associations between higher BMI and income in women (table 3, supplementary table G). Use of genome-wide methods to account for genetic and socioeconomic status correlations between close and distant relatives did not alter our findings (supplementary table H).

## Discussion

Using genetic variants as unconfounded proxies for height and BMI, our study provides evidence that shorter stature and higher BMI lead to lower measures of several aspects of socioeconomic status. It is important to note that our data are consistent with the height and BMI to socioeconomic status associations being only partly causal—we have not excluded a causal effect in the other direction. The study adds causal evidence to a large number of observational studies. This work may have important implications for public health, as low socioeconomic status increases mortality and morbidity.^2^ ^3^ The association between socioeconomic status measures and health was strong in the UK Biobank data, where, for example, people possessing a university degree had a 38% lower odds of coronary artery disease compared with those without degree level education. Our study also showed sex differences in the causal relations between height or BMI and socioeconomic status that are consistent with observational data. Height effects were stronger in men, but the BMI effects tended to be stronger in women.

### Evidence for taller stature leading to higher socioeconomic status

The causal effect, as estimated using genetics, of taller stature on higher socioeconomic status was present in four of the five measures of socioeconomic status. For income, where the statistical evidence was strongest, the estimated causal effects were approximately two to three times stronger in men than in women. The causal evidence for greater height leading to higher levels of socioeconomic status is consistent with observational studies, in which taller stature was associated with higher job class, earnings, and educational attainment.^4^ ^5^ One US based study showed a reduction in earnings of $789 per annum per inch of height. This equates to £1250 per SD (6.3 cm) of height in our study, which is very similar to our genetic estimate of £1130.^13^ Despite the strong evidence that taller stature directly influences measures of socioeconomic status, the genetic estimates were consistently smaller than the observational estimates. These differences indicate that the observed association between taller stature and higher socioeconomic status is a mixture of direct causal effects and other factors that could include a causal effect in the opposite direction.

A range of factors could link taller stature to higher socioeconomic status, although this study did not consider which of these factors were involved. Some of the possibilities include complex interactions between self esteem, stigma, positive discrimination,^13^ and increased intelligence.^4^ ^27^ ^28^ Evidence shows that self esteem, leadership perception, and height discrimination tend to be greater in men than in women, which fits with our findings.^29^ ^30^ ^31^

### Evidence for higher BMI leading to lower socioeconomic status

Higher BMI, as estimated using genetics, was causally associated with having a lower annual household income and higher levels of deprivation. These associations were stronger in women, with no consistent evidence of a causal relation between higher BMI and lower socioeconomic status measures in men. These findings were consistent with previous literature, in which most associations of BMI with socioeconomic status were observed in women only.^7^ ^32^ We found no evidence that the associations between higher BMI and educational outcomes were causal, a result consistent with a review of the effect of BMI on social outcomes.^32^ Our findings add to evidence from observational studies, in which BMI is associated with lower levels of employment, less skilled work, and lower income.^32^ ^33^ A range of factors could link higher BMI to lower income and higher deprivation in women, although this study did not consider which of these factors were involved. One of the possibilities is discrimination in the workplace, with overweight job applicants and employees being evaluated more negatively.^32^ The disparity between the sexes may be partially explained by discrimination, which may occur at lower weight levels for women than for men.^34^ ^35^ Additionally, cultivation theory in social science indicates that very thin women are idealised and more socially valued, compared with their normal weight and overweight peers.^33^ In contrast, a very different set of social standards exists regarding men’s weight, so discrimination based on body size could well be different in men and women.^33^ Two of the strongest measures in women were household income and Townsend deprivation, which are not just specific to the individual but also indicative of partner’s income. However, additional analyses showed that genetically determined higher BMI was associated with lower income both in non-working women with partners and in working women without a partner, suggesting that the associations were not just driven by partner’s income.

### Limitations of study

Although our results are consistent with a direct causal effect of shorter stature and higher BMI on lower socioeconomic status, some qualifications should be considered. Firstly, the UK Biobank participants were born between 1938 and 1971, and the causal associations may not remain in today’s society or be generalisable to societies outside of the United Kingdom. The causal associations may have been influenced by parental genotype-socioeconomic status associations. For example, the causal pathway could reflect parental genetic predisposition to higher BMI resulting in families moving to a more obese and lower socioeconomic status neighbourhood, which in turn could lower children’s socioeconomic status. Because parents’ and children’s genotypes are correlated, this pathway could lead to a genetic association between UK Biobank participants’ socioeconomic status and BMI that reflects parental factors during the 20th century. However, such a pathway would be unlikely to result in genetic associations between BMI and socioeconomic status that were stronger in women than in men.

Secondly, higher BMI leads to poorer health; this could affect productivity, which in turn could affect socioeconomic status. However, we saw evidence of genetic associations between higher BMI and lower socioeconomic status in women reporting no adverse health outcomes as well as in those reporting health problems (supplementary table E). We also need to take care in interpreting negative results; although the large sample size of the UK Biobank provided greater than 95% power for investigating the causal relations between height and socioeconomic status, power was limited for some of the causal associations for BMI. The SNPs selected for height and BMI may have effects on socioeconomic status not mediated by their effects on height or BMI (pleiotropy), which were not measured but could potentially affect socioeconomic status. However, to minimise this possibility, we selected SNPs carefully and used the Egger method, which can detect and adjust for pleiotropy bias in many scenarios^24^ (hence the broader confidence intervals observed).

The educational, job status, and income data used in this study were self reported, which may result in measurement bias. However, Townsend deprivation index was derived by the UK Biobank and we observed consistent trends across the different socioeconomic status constructs, suggesting limited bias due to self report. Socioeconomic status is a very complex multidimensional construct. We looked at a range of individual components and observed similar trends for each, but the selected variables may not cover the entirety of social status. This study used a homogenous population, so the results may not be generalisable to other ethnic groups. We note that the genetic variants associated with BMI together explain only 1.5% of the variation in BMI, but collectively the variants provide a robust test, as reflected by the strong F statistic of the genetic risk score. The 69 genetic variants associated with BMI provided a stronger “instrument” than those used in previous mendelian randomisation studies that inferred a causal effect of higher BMI leading to lower vitamin D concentrations and higher risk of heart failure and markers of poor metabolic health.^36^ ^37^

Finally, height, BMI, and socioeconomic status are subtly stratified across the United Kingdom, with people living and working in the north of the country having lower socioeconomic status, higher BMI, and shorter stature, on average, than those in the south. If genetic variants are also subtly different between north and south, this could have confounded our results. However, several factors mean that this population stratification should not have caused false positive results. Firstly, we would not have expected to have seen differences between men and women (because gene allele frequencies do not differ between the sexes). Secondly, we used both within UK genetic ancestry principal components and a second method that corrects for all levels of relatedness, and our results did not change.

### Conclusion

In summary, using up to 119 000 participants from the UK Biobank, we provide evidence that high BMI and short stature, as estimated by genetics, are causally related to lower socioeconomic status. Further work is needed to understand the factors that lead to and from anthropometric traits and socioeconomic status.

**What is already known on this topic**

Socioeconomic status influences morbidity and mortality, with a recent review highlighting the 18 year gap in life expectancy between men living in the poorest and richest boroughs of London
Taller stature and lower body mass index (BMI) are associated with higher socioeconomic status, but the causal directions of these associations are poorly understood
Understanding the causal directions of these associations is important for public health and wellbeing policies

**What this study adds**

This study provides a high level of evidence (using 119 000 participants from the UK Biobank) for a causal effect from shorter stature and higher BMI to lower measures of socioeconomic status
Shorter height, as estimated by genetics, leads to lower levels of education, lower job status, and less income in men in particular, and higher BMI leads to lower income and greater deprivation in women
Genetic evidence has the advantage of being largely free from the problems that afflict observational studies; analyses using inherited DNA variation are much more robust to confounding, bias, and reverse causality

## Web Extra

Extra material supplied by the author

## Notes

This research has been conducted using the UK Biobank resource.

Ethical approval: UK Biobank has received ethics approval from the National Health Service National Research Ethics Service (ref 11/NW/0382).

Transparency statement: The lead author (the manuscript’s guarantor) affirms that this manuscript is an honest, accurate, and transparent account of the study being reported; that no important aspects of the study have been omitted; and that any discrepancies from the study as planned (and, if relevant, registered) have been explained.

Data sharing: The data reported in this paper are available via application directly to the UK Biobank.
