# Supplementary material for: Height, body mass index, and socioeconomic status: mendelian randomisation study in UK Biobank
Source: BMJ. 2016 Mar 8;352:i582. doi: 10.1136/bmj.i582 (PMC4783516; doi:10.1136/bmj.i582)
Supplement: Supplementary file 1 — Supplementary methods, tables, and figures [file tyrj029564.ww1_default.pdf]

Supplementary Information

Supplementary Methods

UK Biobank Quality Control

The UK Biobank performed extensive quality control on the genetic data including the exclusion of the majority of third degree or closer relatives from a genetic kinship analysis of 96% of individuals. We performed an additional round of principal components analysis on these 120,286 UK Biobank participants. We selected 95,535 independent single nucleotide polymorphisms (pairwise r^2 <0.1) directly genotyped with a minor allele frequency ≥ 2.5% and missingness <1.5% across all UK Biobank participants with genetic data available at the time of this study (*n*=152,732), and with Hardy-Weinberg $P>1x10^{-6}$ within the white British participants. Principal components were subsequently generated using FlashPCA (*1*) and the first five adjusted for in all analyses.

***Power calculation**:* To assess the power of our study, we calculated the approximate number of individuals we would need to detect the expected effect given the gene score – height/BMI associations and the height/BMI-socioeconomic status measure associations: Townsend deprivation index, income, degree level education or job class associations. We used the product of the variance explained by the instrumental variable-height or BMI association and the height or BMI – socioeconomic status associations and a range of P values including 0.05, 0.01 and 0.001 to determine the numbers required to have at least 80% power.

Supplementary table A: Comparison of key demographics for individuals reporting or not reporting each socioeconomic status measure

Demographic variable	Data available	Data missing	P^
**Age completed full time education**			
N	82,543	37,126	
Age at recruitment in years (SD)	57.5 (7.9)	55.6 (7.9)	<1E-15
Male, N (%)	38,342 (46.5)	18,310 (49.3)	<0.001
Mean height in m (SD)	168 (9)	170 (9)	<1E-15
Mean BMI in kgm-2 (SD)	27.9 (4.9)	26.7 (4.5)	<1E-15
**Degree level education**			
N	118,565	1,104	
Age at recruitment in years (SD)	56.9 (7.9)	59.6 (7.5)	<1E-15
Male, N (%)	56,111 (47.3)	541 (49.0)	0.27
Mean height in m (SD)	169 (9)	167 (9)	1.00E-11
Mean BMI in kgm-2 (SD)	27.5 (4.8)	28.2 (5.0)	3.00E-05
**Job class**			
N	76,404	43,265	
Age at recruitment in years (SD)	54.4 (7.6)	61.4 (6.3)	<1E-15
Male, N (%)	37,608 (49.2)	19,044 (44.0)	<0.001
Mean height in m (SD)	170 (9)	167 (9)	<1E-15
Mean BMI in kgm-2 (SD)	27.4 (4.7)	27.7 (5.0)	3.00E-15
**Income**			
N	103,327	16,342	
Age at recruitment in years (SD)	56.5 (8.0)	59.3 (7.4)	<1E-15
Male, N (%)	50,862 (49.2)	5,790 (35.4)	<0.001
Mean height in m (SD)	169 (9)	166 (9)	<1E-15
Mean BMI in kgm-2 (SD)	27.5 (4.8)	27.6 (5.0)	2.00E-05
**Townsend deprivation index**			
N	119,519	150	
Age at recruitment in years (SD)	56.9 (7.9)	55.3 (8.0)	0.015
Male, N (%)	56,582 (47.3)	70 (46.7)	0.87
Mean height in m (SD)	169 (9.2)	169 (8.9)	0.51
Mean BMI in kgm-2 (SD)	27.5 (4.8)	27.8 (5.4)	0.43

^ P values represent age and sex adjusted comparisons of the two groups of individuals with and without data available.

Supplementary table B: Associations between the 5 socioeconomic status markers and a range of health outcomes in the UK Biobank

Disease	socioeconomic status measure	N cases (controls)	Odds ratio for disease in the UK Biobank per SD higher socioeconomic status (95%CI)^	p
Coronary artery disease	Age completed full time education	4670 (77890)	0.85 (0.82, 0.88)	7.00E-25
Hypertension		47915 (34151)	0.96 (0.94, 0.97)	3.00E-08
Long illness		28515 (51982)	0.91 (0.89, 0.92)	8.00E-37
Type 2 diabetes		3144 (77544)	1.00 (0.96, 1.03)	8.10E-01
Coronary artery disease	Degree	5663 (112921)	0.62 (0.59, 0.66)	1.00E-55
Hypertension		64881 (53040)	0.81 (0.79, 0.83)	9.00E-63
Long illness		38872 (77100)	0.87 (0.85, 0.89)	3.00E-27
Type 2 diabetes		3958 (112185)	0.81 (0.76, 0.87)	3.00E-09
Coronary artery disease	Job class	2280 (74133)	0.71 (0.65, 0.78)	6.00E-14
Hypertension		37446 (38507)	0.80 (0.77, 0.83)	1.00E-34
Long illness		20712 (54147)	0.86 (0.83, 0.89)	2.00E-16
Type 2 diabetes		1914 (73217)	0.98 (0.89, 1.08)	6.90E-01
Coronary artery disease	Income	4778 (98563)	0.73 (0.71, 0.76)	2.00E-76
Hypertension		55864 (46947)	0.93 (0.91, 0.94)	5.00E-28
Long illness		33431 (67856)	0.74 (0.73, 0.75)	<1E-15
Type 2 diabetes		3420 (97860)	0.83 (0.80, 0.86)	6.00E-23
Coronary artery disease	Townsend deprivation index	5752 (113786)	0.78 (0.76, 0.80)	4.00E-68
Hypertension		65499 (53314)	0.96 (0.95, 0.98)	5.00E-09
Long illness		39239 (77617)	0.80 (0.79, 0.81)	2.00E-158
Type 2 diabetes		3998 (113062)	0.86 (0.83, 0.89)	9.00E-19

^ adjusted for age, sex and BMI

Supplementary table C: Summary of the body mass index (BMI) and height variants previously identified as associated with those traits at genome wide significance

**Trait**	**Genetic variant**	**Locus**	**Exclude from score**	**Reason for exclusion**	**Trait raising allele**	**Trait lowering allele**	**Directly genotyped or Imputed**	**Imputation quality**	**Beta representing SD change in BMI or height for each SNP in UK Biobank data**	**P value**
BMI	rs1000940	*RABEP1*	No	NA	G	A	Imputed	0.99624	0.011 (0.004)	1.60E-02
BMI	rs10132280	*STXBP6*	No	NA	C	A	Imputed	0.97496	0.020 (0.005)	1.10E-05
BMI	rs1016287	*FLJ30838*	No	NA	T	C	Imputed	0.99411	0.019 (0.004)	2.00E-05
BMI	rs10182181	*ADCY3*	No	NA	G	A	Imputed	0.99521	0.033 (0.004)	1.40E-15
BMI	rs10733682	*LMX1B*	No	NA	A	G	Imputed	0.9576	0.019 (0.004)	5.90E-06
BMI	rs10938397	*GNPDA2*	No	NA	G	A	Imputed	1	0.030 (0.004)	5.80E-13
BMI	rs10968576	*LINGO2*	No	NA	G	A	Imputed	1	0.024 (0.004)	6.90E-08
BMI	rs11030104	*BDNF*	Yes	BMI-raising allele also associated with regular smoking (which itself has a causal effect on BMI in opposite direction)	A	G	Imputed	0.99931	NA	NA
BMI	rs11057405	*CLIP1*	No	NA	G	A	Imputed	1	0.030 (0.007)	4.70E-06
BMI	rs11126666	*KCNK3*	No	NA	A	G	Imputed	0.99485	0.002 (0.005)	7.10E-01
BMI	rs11165643	*PTBP2*	No	NA	T	C	Imputed	0.99575	0.016 (0.004)	9.50E-05
BMI	rs11191560	*NT5C2*	No	NA	C	T	Imputed	0.99989	0.026 (0.008)	6.50E-04

BMI	rs11583200	*ELAVL4*	No	NA	C	T	Imputed	0.98728	0.019 (0.004)	7.70E-06
BMI	rs1167827	*HIP1*	No	NA	G	A	Imputed	1	0.020 (0.004)	1.80E-06
BMI	rs11688816	*EHBP1*	No	NA	G	A	Imputed	0.98096	0.014 (0.004)	9.40E-04
BMI	rs11727676	*HHIP*	No	NA	T	C	Imputed	1	-0.003 (0.007)	6.60E-01
BMI	rs11847697	*PRKD1*	No	NA	T	C	Imputed	1	0.014 (0.010)	1.70E-01
BMI	rs12286929	*CADM1*	No	NA	G	A	Imputed	0.99124	0.010 (0.004)	1.20E-02
BMI	rs12401738	*FUBP1*	No	NA	A	G	Imputed	0.99528	0.012 (0.004)	3.30E-03
BMI	rs12429545	*OLFM4*	No	NA	A	G	Imputed	0.97759	0.027 (0.006)	8.00E-06
BMI	rs12446632	*GPRC5B*	No	NA	G	A	Imputed	0.99978	0.028 (0.006)	2.40E-06
BMI	rs12566985	*FPGT-TNNI3K*	No	NA	G	A	Imputed	0.9947	0.011 (0.004)	6.10E-03
BMI	rs12885454	*PRKD1*	No	NA	C	A	Imputed	0.99569	0.015 (0.004)	4.60E-04
BMI	rs12940622	*RPTOR*	No	NA	G	A	Imputed	0.99796	0.017 (0.004)	5.90E-05
BMI	rs13021737	*TMEM18*	No	NA	G	A	Imputed	0.99072	0.059 (0.005)	9.10E-27
BMI	rs13078960	*CADM2*	No	NA	G	T	Imputed	0.9915	0.024 (0.005)	2.50E-06
BMI	rs13107325	*SLC39A8*	Yes	Missense Ala/Thr polymorphism located in exon 7 of SLC39A8, which encodes a zinc transporter that also transports cadmium and manganese. It is also associated with BP and HDL levels,	T	C	Imputed	1	NA	NA

				and presumably these and the BMI effect are secondary to the metal ion transport variation.						
BMI	rs13191362	*PARK2*	No	NA	A	G	Imputed	0.98973	0.026 (0.006)	3.10E-05
BMI	rs1516725	*ETV5*	No	NA	C	T	Imputed	0.99495	0.032 (0.006)	1.00E-07
BMI	rs1528435	*UBE2E3*	No	NA	T	C	Imputed	0.99738	0.014 (0.004)	6.60E-04
BMI	rs1558902	*FTO*	No	NA	A	T	Imputed	0.99914	0.077 (0.004)	1.50E-75
BMI	rs16851483	*RASA2*	No	NA	T	G	Imputed	0.99906	0.028 (0.008)	6.80E-04
BMI	rs16951275	*MAP2K5*	No	NA	T	C	Imputed	0.99819	0.032 (0.005)	4.40E-11
BMI	rs17001654	*SCARB2*	Yes	SNP not in Hardy-Weinberg equilibrium	G	C	Imputed	0.9483	NA	NA
BMI	rs17024393	*GNAT2*	No	NA	C	T	Imputed	0.98934	0.074 (0.013)	1.20E-08
BMI	rs17094222	*HIF1AN*	No	NA	C	T	Imputed	0.96874	0.013 (0.005)	8.50E-03
BMI	rs17405819	*HNF4G*	No	NA	T	C	Imputed	0.99793	0.014 (0.004)	1.30E-03
BMI	rs17724992	*PGPEP1*	No	NA	A	G	Imputed	0.98342	0.023 (0.005)	1.10E-06
BMI	rs1808579	*C18orf8*	No	NA	C	T	Imputed	0.99797	0.022 (0.004)	1.50E-07
BMI	rs1928295	*TLR4*	No	NA	T	C	Imputed	0.99998	0.010 (0.004)	1.60E-02
BMI	rs2033529	*TDRG1*	Yes	SNP not available	G	A	NA	NA	NA	NA
BMI	rs2033732	*RALYL*	No	NA	C	T	Imputed	1	0.002 (0.005)	6.70E-01
BMI	rs205262	*C6orf106*	No	NA	G	A	Imputed	0.9968	0.028 (0.005)	1.10E-09
BMI	rs2075650	*TOMM40*	Yes	SNP not in Hardy-Weinberg equilibrium	A	G	Imputed	0.9865	NA	NA
BMI	rs2112347	*POC5*	No	NA	T	G	Imputed	1	0.026	6.30E-10

									(0.004)	
BMI	rs2121279	*LRP1B*	No	NA	T	C	Imputed	0.98723	0.006 (0.006)	3.70E-01
BMI	rs2176598	*HSD17B12*	No	NA	T	C	Imputed	1	0.023 (0.005)	1.30E-06
BMI	rs2207139	*TFAP2B*	No	NA	G	A	Imputed	0.9989	0.038 (0.005)	1.80E-12
BMI	rs2245368	*PMS2L11*	No	NA	C	T	Imputed	1	0.022 (0.005)	8.00E-05
BMI	rs2287019	*QPCTL*	No	NA	C	T	Imputed	0.97852	0.035 (0.005)	1.00E-10
BMI	rs2365389	*FHIT*	No	NA	C	T	Imputed	0.99305	0.029 (0.004)	2.70E-12
BMI	rs2650492	*SBK1*	No	NA	A	G	Imputed	0.98144	0.019 (0.005)	3.60E-05
BMI	rs2820292	*NAV1*	No	NA	C	A	Imputed	1	0.019 (0.004)	3.60E-06
BMI	rs29941	*KCTD15*	No	NA	G	A	Imputed	1	0.018 (0.004)	5.00E-05
BMI	rs3101336	*NEGR1*	No	NA	C	T	Imputed	1	0.027 (0.004)	9.50E-11
BMI	rs3736485	*DMXL2*	No	NA	A	G	Imputed	0.98728	0.011 (0.004)	6.40E-03
BMI	rs3810291	*ZC3H4*	No	NA	A	G	Imputed	1	0.028 (0.004)	1.80E-10
BMI	rs3817334	*MTCH2*	No	NA	T	C	Imputed	1	0.031 (0.004)	1.40E-13
BMI	rs3849570	*GBE1*	No	NA	A	C	Imputed	0.99509	0.011 (0.004)	7.80E-03
BMI	rs3888190	*ATP2A1*	Yes	Associated with lots of other traits and is a big haplotype	A	C	Imputed	0.99808	NA	NA
BMI	rs4256980	*TRIM66*	No	NA	G	C	Imputed	0.99283	0.021 (0.004)	1.70E-06
BMI	rs4740619	*C9orf93*	No	NA	T	C	Imputed	0.99762	0.017 (0.004)	5.70E-05
BMI	rs543874	*SEC16B*	No	NA	G	A	Imputed	1	0.049 (0.005)	3.40E-22

BMI	rs6477694	*EPB41L4B*	No	NA	C	T	Imputed	0.99022	0.008 (0.004)	6.70E-02
BMI	rs6567160	*MC4R*	No	NA	C	T	Imputed	0.99663	0.054 (0.005)	9.50E-29
BMI	rs657452	*AGBL4*	No	NA	A	G	Imputed	0.98709	0.014 (0.004)	8.40E-04
BMI	rs6804842	*RARB*	No	NA	G	A	Imputed	0.98778	0.009 (0.004)	3.20E-02
BMI	rs7138803	*BCDIN3D*	No	NA	A	G	Imputed	1	0.034 (0.004)	1.30E-15
BMI	rs7141420	*NRXN3*	No	NA	T	C	Imputed	0.98379	0.019 (0.004)	6.70E-06
BMI	rs7243357	*GRP*	No	NA	T	G	Imputed	0.98998	0.012 (0.005)	2.10E-02
BMI	rs758747	*NLRC3*	No	NA	T	C	Imputed	0.97187	0.014 (0.005)	2.00E-03
BMI	rs7599312	*ERBB4*	No	NA	G	A	Imputed	0.97294	0.019 (0.005)	3.60E-05
BMI	rs7899106	*GRID1*	No	NA	G	A	Imputed	0.98612	0.023 (0.009)	1.40E-02
BMI	rs9400239	*FOXO3*	No	NA	C	T	Imputed	0.99206	0.017 (0.005)	2.30E-04
BMI	rs9581854	*MTIF3*	No	NA	T	C	Imputed	0.98643	0.015 (0.005)	6.20E-03
BMI	rs9925964	*KAT8*	Yes	SNP not in Hardy-Weinberg equilibrium	A	G	Imputed	1		
Height	rs10083886	*SOX9*	No	NA	T	C	Imputed	0.96954	0.021 (0.005)	1E-05
Height	rs10131337	*PAX9*	No	NA	T	C	Imputed	0.98303	0.026 (0.005)	6E-08
Height	rs10152739	*SPRED1*	No	NA	T	A	Imputed	0.9879	0.016 (0.005)	7E-04
Height	rs1036477	*FBN1*	No	NA	A	G	Imputed	0.9957	0.029 (0.007)	2E-05
Height	rs1036821	*ZFAT*	No	NA	G	A	Imputed	0.97861	0.042 (0.004)	1E-20
Height	rs1047014	*ID4*	No	NA	C	T	Imputed	1	0.028	4E-09

									(0.005)	
Height	rs1055144	*NFE2L3*	No	NA	T	C	Imputed	1	0.029 (0.005)	2E-08
Height	rs1074683	*PXMP4*	No	NA	C	G	Imputed	0.99541	0.042 (0.005)	4E-19
Height	rs10748128	*FRS2*	No	NA	T	G	Imputed	1	0.032 (0.004)	6E-14
Height	rs10767838	*C11orf46*	No	NA	A	G	Imputed	0.99185	0.011 (0.005)	2E-02
Height	rs10770705	*SLCO1C1*	No	NA	A	C	Imputed	1	0.022 (0.004)	3E-07
Height	rs10779751	*FRAP1*	No	NA	A	G	Imputed	1	0.017 (0.005)	2E-04
Height	rs10780910	*SPIN1*	No	NA	T	A	Imputed	0.98414	0.028 (0.004)	2E-11
Height	rs10790381	*ARHGEF12*	No	NA	A	G	Imputed	0.99606	0.029 (0.005)	9E-08
Height	rs10794175	*FAM53B*	No	NA	T	G	Imputed	0.99344	0.018 (0.004)	9E-06
Height	rs10863936	*DTL*	No	NA	G	A	Imputed	1	0.019 (0.004)	4E-06
Height	rs10877030	*CTDSP2*	No	NA	T	G	Imputed	0.98828	0.033 (0.004)	2E-14
Height	rs10880969	*SLC38A2*	No	NA	C	T	Imputed	0.99792	0.029 (0.004)	6E-11
Height	rs10883563	*FAM178A*	No	NA	A	C	Imputed	1	0.024 (0.004)	3E-09
Height	rs10948222	*SUPT3H*	No	NA	C	T	Imputed	0.99409	0.014 (0.004)	6E-04
Height	rs10995319	*PRKG1*	No	NA	T	C	Imputed	0.9964	0.019 (0.005)	1E-04
Height	rs10997979	*MYPN*	No	NA	G	A	Imputed	0.99812	0.030 (0.004)	3E-13
Height	rs11047239	*SOX5*	No	NA	G	C	Imputed	0.99285	0.025 (0.004)	1E-08
Height	rs11049611	*CCDC91*	No	NA	C	T	Imputed	0.99616	0.041 (0.004)	1E-20
Height	rs1113765	*SEPT14*	No	NA	G	A	Imputed	0.98428	0.015	6E-03

									(0.005)	
Height	rs11144688	*PCSK5*	No	NA	G	A	Imputed	1	0.051 (0.006)	4E-16
Height	rs11152213	*MC4R*	No	NA	C	A	Imputed	0.99902	0.034 (0.005)	3E-12
Height	rs11156098	*ARID1B*	No	NA	T	C	Imputed	0.97473	0.024 (0.007)	4E-04
Height	rs11221442	*FLI1*	No	NA	G	C	Imputed	0.99462	-0.005 (0.005)	3E-01
Height	rs1155939	*C6orf173*	No	NA	A	C	Imputed	0.99833	0.048 (0.004)	2E-32
Height	rs11612228	*B4GALNT3*	No	NA	T	C	Imputed	0.96996	0.036 (0.004)	1E-16
Height	rs11616067	*MED13L*	No	NA	A	G	Imputed	1	0.018 (0.005)	1E-04
Height	rs11616380	*SPRY2*	No	NA	T	G	Imputed	0.98577	0.014 (0.005)	3E-03
Height	rs11618507	*SLC7A1*	No	NA	T	G	Imputed	0.99005	0.013 (0.005)	8E-03
Height	rs11624136	*DAAM1*	No	NA	A	G	Imputed	0.99632	0.013 (0.004)	1E-03
Height	rs11640018	*CFDP1*	No	NA	C	T	Imputed	1	0.010 (0.004)	1E-02
Height	rs11642612	*FLJ25404*	No	NA	C	A	Imputed	0.99914	0.017 (0.004)	4E-05
Height	rs11648796	*NARFL*	No	NA	G	A	Imputed	0.90086	0.045 (0.005)	3E-18
Height	rs11659752	*NFATC1*	No	NA	T	G	Imputed	0.99007	0.022 (0.004)	1E-06
Height	rs11683207	*ZAP70*	Yes	Imputation r2<0.9	T	C	NA	NA	NA	NA
Height	rs11684404	*EIF2AK3*	No	NA	C	T	Imputed	0.99855	0.036 (0.004)	1E-16
Height	rs11687941	*HDLBP*	No	NA	C	G	Imputed	0.99945	0.021 (0.005)	1E-05
Height	rs1171615	*SLC16A9*	No	NA	C	T	Imputed	0.99323	0.031 (0.005)	1E-10
Height	rs11750568	*ADAMTS2*	No	NA	A	G	Imputed	0.99518	0.016 (0.004)	2E-04

Height	rs11783655	*PLEC1*	No	NA	T	A	Imputed	0.98049	0.031 (0.004)	2E-13
Height	rs11799609	*SDCCAG8*	No	NA	T	G	Imputed	0.98286	0.012 (0.006)	4E-02
Height	rs11835818	*BCL7A*	No	NA	C	T	Imputed	0.98771	0.015 (0.004)	2E-04
Height	rs11855014	*PDE8A*	No	NA	G	A	Imputed	0.97241	0.013 (0.004)	4E-03
Height	rs11867479	*KCNJ16*	No	NA	T	C	Imputed	1	0.030 (0.004)	3E-12
Height	rs11880992	*DOT1L*	No	NA	A	G	Imputed	0.99401	0.039 (0.004)	2E-20
Height	rs1199734	*LATS2*	No	NA	G	T	Imputed	0.99442	0.028 (0.005)	2E-07
Height	rs12120956	*CAPZA1*	No	NA	G	A	Imputed	1	0.021 (0.005)	2E-05
Height	rs12137162	*CAPZB*	No	NA	A	C	Imputed	1	0.022 (0.005)	2E-06
Height	rs12186664	*PCSK1*	No	NA	T	A	Imputed	0.9915	0.013 (0.004)	2E-03
Height	rs12190423	*OGFRL1*	No	NA	G	C	Imputed	0.98213	0.016 (0.004)	3E-04
Height	rs12209223	*FILIP1*	No	NA	A	C	Imputed	0.98097	0.046 (0.007)	2E-11
Height	rs12214804	*HMGA1*	No	NA	C	T	Imputed	0.99476	0.091 (0.007)	3E-36
Height	rs12323101	*PDS5B*	No	NA	A	G	Imputed	0.99874	0.018 (0.004)	2E-05
Height	rs12330322	*RYBP*	No	NA	C	T	Imputed	0.99412	0.034 (0.005)	8E-12
Height	rs1233627	*TRIM27*	No	NA	T	C	Imputed	0.99851	0.023 (0.004)	3E-08
Height	rs12435366	*NFKBIA*	No	NA	C	T	Imputed	0.98427	0.010 (0.005)	5E-02
Height	rs12470505	*CCDC108*	No	NA	T	G	Imputed	1	0.048 (0.007)	3E-12
Height	rs12474201	*SOCS5*	No	NA	A	G	Imputed	1	0.032 (0.004)	1E-13

Height	rs12513181	*NUDT6*	No	NA	C	A	Imputed	0.99914	0.020 (0.005)	1E-05
Height	rs12519505	*AP3B1*	No	NA	C	T	Imputed	0.99272	0.022 (0.005)	6E-06
Height	rs12538407	*IGF2BP3*	No	NA	A	G	Imputed	0.99068	0.036 (0.004)	6E-18
Height	rs12639764	*TET2*	No	NA	T	C	Imputed	0.98841	0.031 (0.004)	3E-13
Height	rs12669267	*WBSCR28*	No	NA	C	T	Imputed	0.97258	0.028 (0.006)	7E-06
Height	rs12693589	*STAT1*	No	NA	C	T	Imputed	0.98669	0.021 (0.005)	9E-06
Height	rs12779328	*CCDC3*	No	NA	C	T	Imputed	0.99272	0.035 (0.005)	1E-14
Height	rs12855	*CDKN2C*	No	NA	T	C	Imputed	1	0.060 (0.007)	4E-17
Height	rs12882130	*MARK3*	No	NA	C	G	Imputed	0.95464	0.024 (0.004)	1E-08
Height	rs12904334	*ARIH1*	No	NA	A	G	Imputed	1	0.068 (0.017)	7E-05
Height	rs12987566	*METTL8*	No	NA	T	C	Imputed	0.99346	0.026 (0.005)	4E-08
Height	rs13006748	*WDR35*	No	NA	C	G	Imputed	0.96536	0.003 (0.005)	6E-01
Height	rs13088462	*DOCK3*	No	NA	C	T	Imputed	1	0.060 (0.009)	2E-10
Height	rs13113518	*CLOCK*	No	NA	C	T	Imputed	0.99849	0.016 (0.004)	2E-04
Height	rs13150868	*ESSPL*	No	NA	T	G	Imputed	0.99747	0.012 (0.004)	4E-03
Height	rs13177718	*FER*	No	NA	C	T	Imputed	1	0.031 (0.008)	7E-05
Height	rs1325596	*PAPPA2*	No	NA	A	G	Imputed	1	0.029 (0.004)	3E-12
Height	rs1326023	*MC3R*	No	NA	A	G	Imputed	0.98457	0.012 (0.005)	8E-03
Height	rs13388725	*GCC2*	No	NA	G	A	Imputed	0.99389	0.008 (0.004)	7E-02

Height	rs13416119	*EML4*	No	NA	A	G	Imputed	0.98764	0.024 (0.007)	9E-04
Height	rs1401795	*C17orf67*	Yes	SNP not in Hardy-Weinberg equilibrium	A	G	NA	NA	NA	NA
Height	rs1405212	*VGLL2*	No	NA	C	T	Imputed	0.9969	0.027 (0.004)	2E-10
Height	rs14062	*MIB1*	No	NA	G	A	Imputed	0.99528	0.009 (0.004)	5E-02
Height	rs1420023	*CDKN1B*	Yes	SNP not available	C	G	NA	NA	NA	NA
Height	rs143384	*GDF5*	No	NA	G	A	Imputed	1	0.092 (0.004)	1E-109
Height	rs1461503	*BSX*	No	NA	C	A	Imputed	0.99729	0.021 (0.004)	4E-07
Height	rs1546391	*ZBTB20*	No	NA	G	C	Imputed	0.98751	0.031 (0.008)	1E-04
Height	rs1550162	*EIF3H*	No	NA	G	A	Imputed	0.97822	0.022 (0.005)	3E-06
Height	rs1552173	*PSCD1*	No	NA	C	T	Imputed	0.98704	0.013 (0.004)	1E-03
Height	rs1562975	*RPL34*	No	NA	A	G	Imputed	0.99896	0.027 (0.004)	2E-09
Height	rs1576900	*ADAMTSL1*	No	NA	G	A	Imputed	0.97217	0.011 (0.005)	2E-02
Height	rs1582931	*CCDC100*	No	NA	G	A	Imputed	1	0.023 (0.004)	2E-08
Height	rs1599473	*NOV*	No	NA	G	T	Imputed	0.98821	0.030 (0.005)	7E-10
Height	rs1614303	*FGFR2*	No	NA	T	G	Imputed	0.99718	0.015 (0.005)	6E-03
Height	rs165189	*PSD2*	No	NA	G	A	Imputed	0.9813	0.014 (0.006)	2E-02
Height	rs1658351	*FLNB*	No	NA	C	T	Imputed	0.99121	0.024 (0.004)	2E-08
Height	rs1659127	*MKL2*	No	NA	A	G	Imputed	1	0.022 (0.004)	2E-07
Height	rs1681630	*PTPRJ*	No	NA	T	C	Imputed	0.99573	0.024 (0.004)	2E-08
Height	rs16834765	*PTP4A2*	No	NA	T	C	Imputed	1	0.051	6E-09

									(0.009)	
Height	rs16895130	*CCND3*	No	NA	G	A	Imputed	0.98958	0.024 (0.005)	3E-07
Height	rs16964211	*CYP19A1*	No	NA	G	A	Imputed	1	0.043 (0.010)	1E-05
Height	rs16968242	*SCAPER*	No	NA	G	C	Imputed	0.99962	0.035 (0.008)	3E-05
Height	rs17038954	*PXDN*	No	NA	T	C	Imputed	0.95649	0.029 (0.009)	7E-04
Height	rs17081935	*C4orf14*	No	NA	T	C	Imputed	0.9998	0.035 (0.005)	2E-11
Height	rs17113369	*RWDD3*	No	NA	T	C	Imputed	1	0.009 (0.012)	5E-01
Height	rs17122659	*SLC16A7*	No	NA	G	A	Imputed	0.99103	0.023 (0.007)	5E-04
Height	rs17250196	*GATS/PVRIG*	No	NA	T	G	Imputed	0.96925	0.035 (0.009)	1E-04
Height	rs17264185	*SMAD6*	No	NA	G	A	Imputed	0.99628	0.026 (0.005)	6E-08
Height	rs17330192	*FAM8A1*	No	NA	C	T	Imputed	0.98161	-0.004 (0.005)	4E-01
Height	rs17349981	*MEX3B*	No	NA	A	T	Imputed	0.99669	0.012 (0.006)	4E-02
Height	rs17391694	*GIPC2*	No	NA	T	C	Imputed	1	0.036 (0.006)	5E-10
Height	rs17410035	*C5orf22*	No	NA	T	G	Imputed	0.99922	-0.006 (0.004)	2E-01
Height	rs17450430	*STAU1*	No	NA	T	A	Imputed	0.9959	0.044 (0.005)	2E-20
Height	rs17511102	*CDC42EP3*	No	NA	T	A	Imputed	1	0.047 (0.007)	3E-11
Height	rs17556750	*PRKG2*	No	NA	A	C	Imputed	0.99479	0.042 (0.005)	8E-21
Height	rs17574650	*GHR*	No	NA	C	A	Imputed	1	0.043 (0.007)	3E-10
Height	rs17783015	*ATP2B1*	No	NA	C	T	Imputed	1	0.009 (0.006)	1E-01
Height	rs17792664	*CHD8*	No	NA	G	C	Imputed	1	0.025	8E-06

									(0.006)	
Height	rs17806888	*SUCLG2*	No	NA	T	C	Imputed	1	0.030 (0.006)	2E-06
Height	rs17807185	*RSBN1L*	No	NA	G	A	Imputed	0.9897	0.011 (0.004)	1E-02
Height	rs1797625	*C3orf17*	No	NA	T	A	Imputed	0.9765	0.021 (0.004)	2E-06
Height	rs1812175	*HHIP*	No	NA	G	A	Imputed	1	0.087 (0.005)	3E-57
Height	rs181338	*ZCCHC6*	No	NA	T	C	Imputed	0.99635	0.035 (0.004)	9E-18
Height	rs1832871	*TULP4*	No	NA	A	G	Imputed	0.99881	0.025 (0.004)	6E-09
Height	rs1884897	*BMP2*	No	NA	A	G	Imputed	0.9921	0.055 (0.004)	4E-38
Height	rs1923367	*ZCCHC24*	No	NA	G	C	Imputed	0.98201	0.036 (0.004)	6E-18
Height	rs1935157	*HLX*	No	NA	G	C	Imputed	1	0.018 (0.005)	9E-05
Height	rs1950500	*NFATC4*	No	NA	T	C	Imputed	1	0.027 (0.004)	1E-09
Height	rs1966913	*LRRC36*	No	NA	A	T	Imputed	0.99972	0.058 (0.010)	8E-09
Height	rs1980850	*RAD51L1*	No	NA	G	A	Imputed	0.99858	0.017 (0.006)	2E-03
Height	rs1996422	*FRYL*	No	NA	G	A	Imputed	0.98043	-0.007 (0.005)	1E-01
Height	rs2013265	*ADAM28*	No	NA	C	T	Imputed	1	0.028 (0.005)	2E-09
Height	rs2023693	*DCUN1D3*	No	NA	G	A	Imputed	0.99918	0.008 (0.004)	5E-02
Height	rs2034172	*WNT5A*	No	NA	G	A	Imputed	0.99308	0.011 (0.004)	1E-02
Height	rs2057291	*GNAS*	No	NA	A	G	Imputed	1	0.019 (0.004)	9E-06
Height	rs2058092	*NUMB*	No	NA	T	C	Imputed	0.98608	0.011 (0.004)	9E-03
Height	rs2072268	*ARSG*	No	NA	G	A	Imputed	1	0.014	7E-04

									(0.004)	
Height	rs2074977	*NFIC*	No	NA	C	A	Imputed	1	0.024 (0.004)	1E-08
Height	rs2079795	*C17orf82*	No	NA	T	C	Imputed	1	0.048 (0.004)	1E-28
Height	rs2093210	*C14orf39*	No	NA	C	T	Imputed	0.97693	0.038 (0.004)	1E-19
Height	rs2117563	*GRB2*	No	NA	G	A	Imputed	0.99931	0.023 (0.006)	2E-05
Height	rs2120335	*PPP3R1*	No	NA	G	A	Imputed	0.99937	0.018 (0.004)	1E-05
Height	rs2123731	*UHRF1*	No	NA	A	G	Imputed	1	0.032 (0.005)	3E-12
Height	rs212524	*ECE1*	No	NA	C	T	Imputed	1	0.022 (0.004)	1E-07
Height	rs2145357	*NT5DC1*	No	NA	G	A	Imputed	0.99659	0.019 (0.005)	4E-05
Height	rs2149163	*BNC2*	No	NA	C	G	Imputed	0.99152	0.017 (0.004)	5E-05
Height	rs2164747	*HSP90B1*	No	NA	G	A	Imputed	0.99925	0.027 (0.007)	3E-05
Height	rs2166898	*GLI2*	No	NA	G	A	Imputed	1	0.034 (0.005)	4E-10
Height	rs217181	*HPR*	No	NA	T	C	Imputed	1	0.021 (0.005)	7E-05
Height	rs2175513	*FAM19A1*	No	NA	G	A	Imputed	0.98947	0.000 (0.004)	1E+00
Height	rs2211866	*KCNJ15*	No	NA	A	G	Imputed	0.98188	0.037 (0.004)	4E-18
Height	rs2224538	*MAFB*	No	NA	T	C	Imputed	0.98816	0.020 (0.004)	4E-06
Height	rs2237886	*KCNQ1*	No	NA	T	C	Imputed	1	0.063 (0.007)	4E-21
Height	rs2272566	*PSMD13*	No	NA	A	G	Imputed	0.99421	-0.007 (0.004)	9E-02
Height	rs2275325	*ZC3H11A*	No	NA	C	G	Imputed	1	0.019 (0.005)	4E-05
Height	rs2280470	*ACAN*	No	NA	A	G	Imputed	1	0.044	3E-24

									(0.004)	
Height	rs2284746	*MFAP2*	No	NA	G	C	Imputed	1	0.036 (0.004)	2E-18
Height	rs2289195	*DNMT3A*	No	NA	A	G	Imputed	1	0.044 (0.004)	6E-26
Height	rs2298265	*ZNF687*	No	NA	C	T	Imputed	1	0.014 (0.007)	3E-02
Height	rs2302580	*CPZ*	No	NA	C	T	Imputed	0.98211	0.025 (0.004)	1E-09
Height	rs2306596	*RFC1*	No	NA	A	C	Imputed	0.99585	0.018 (0.004)	2E-05
Height	rs2306694	*CS*	No	NA	G	A	Imputed	0.99922	0.042 (0.008)	4E-07
Height	rs2326458	*ZDHHC7*	No	NA	C	A	Imputed	1	0.024 (0.005)	3E-07
Height	rs2338115	*PIP4K2B*	No	NA	T	C	Imputed	0.99426	0.027 (0.004)	6E-11
Height	rs2345835	*RDH14*	No	NA	C	T	Imputed	0.99246	0.003 (0.004)	4E-01
Height	rs2510396	*GAL*	No	NA	C	G	Imputed	0.99288	0.041 (0.006)	2E-13
Height	rs2581830	*RFT1*	No	NA	T	C	Imputed	0.99506	0.032 (0.004)	1E-14
Height	rs2597513	*HDAC11*	No	NA	C	T	Imputed	1	0.033 (0.007)	1E-06
Height	rs26024	*FBN2*	No	NA	C	A	Imputed	0.98519	0.014 (0.004)	8E-04
Height	rs2631676	*PCGF5*	No	NA	G	A	Imputed	0.99202	0.039 (0.005)	1E-13
Height	rs2633761	*ITPR1*	No	NA	A	G	Imputed	0.95603	0.010 (0.004)	2E-02
Height	rs2662027	*MIER3*	No	NA	G	T	Imputed	1	0.025 (0.007)	2E-04
Height	rs2682587	*XRCC1*	No	NA	A	C	Imputed	0.99138	0.029 (0.005)	3E-08
Height	rs26868	*CASKIN1*	No	NA	A	T	Imputed	0.99848	0.028 (0.004)	8E-12
Height	rs2715094	*GRB10*	No	NA	G	A	Imputed	0.98915	0.020	3E-05

									(0.005)	
Height	rs273945	*CREB3L2*	No	NA	C	A	Imputed	0.94152	0.021 (0.004)	1E-06
Height	rs2748483	*GRM1*	No	NA	A	T	Imputed	0.99855	0.016 (0.004)	9E-05
Height	rs2763273	*SMOC2*	No	NA	C	T	Imputed	0.99591	0.034 (0.005)	3E-12
Height	rs2781373	*MAX*	No	NA	G	A	Imputed	0.99932	0.028 (0.004)	3E-11
Height	rs2806561	*LUZP1*	No	NA	A	G	Imputed	1	0.019 (0.004)	4E-06
Height	rs2811594	*FAM69A*	No	NA	G	A	Imputed	1	0.014 (0.004)	8E-04
Height	rs2815379	*SLC35D1*	No	NA	G	A	Imputed	1	0.014 (0.005)	2E-03
Height	rs2829941	*APP*	No	NA	T	G	Imputed	0.99247	0.002 (0.004)	7E-01
Height	rs2834442	*KCNE2*	No	NA	A	T	Imputed	1	0.018 (0.004)	2E-05
Height	rs2854207	*CSH2*	No	NA	G	C	Imputed	0.99608	0.053 (0.005)	7E-31
Height	rs2856321	*ETV6*	No	NA	G	A	Imputed	1	0.029 (0.004)	1E-11
Height	rs2871865	*IGF1R*	No	NA	C	G	Imputed	1	0.063 (0.006)	4E-23
Height	rs2888893	*C12orf23*	No	NA	C	T	Imputed	0.99863	0.013 (0.004)	1E-03
Height	rs291979	*GRK5*	No	NA	A	G	Imputed	0.9937	0.022 (0.005)	7E-06
Height	rs2956605	*CRISPLD1*	No	NA	A	C	Imputed	0.97906	0.027 (0.004)	2E-10
Height	rs2961830	*ISL1*	No	NA	A	T	Imputed	0.99448	0.017 (0.004)	8E-05
Height	rs2974438	*SLIT3*	No	NA	G	A	Imputed	0.98375	0.037 (0.005)	2E-13
Height	rs3014219	*AKR1A1*	No	NA	G	A	Imputed	1	0.013 (0.004)	1E-03
Height	rs301901	*NIPBL*	No	NA	A	G	Imputed	0.99547	0.028	3E-11

									(0.004)	
Height	rs310421	*FAM46A*	No	NA	T	G	Imputed	0.99828	0.031 (0.004)	3E-14
Height	rs3116168	*DIS3L2*	No	NA	C	T	Imputed	0.99802	0.046 (0.005)	8E-24
Height	rs3118905	*DLEU7*	No	NA	G	A	Imputed	1	0.056 (0.005)	6E-35
Height	rs3132297	*RXRA*	No	NA	G	A	Imputed	1	0.005 (0.006)	4E-01
Height	rs314263	*LIN28B*	No	NA	C	T	Imputed	0.99833	0.048 (0.004)	3E-28
Height	rs316618	*LTK*	No	NA	T	A	Imputed	0.95894	0.015 (0.005)	4E-03
Height	rs318095	*ATP5G1*	No	NA	T	C	Imputed	0.99849	0.034 (0.004)	1E-16
Height	rs32855	*FAM151B*	No	NA	A	G	Imputed	0.9916	0.019 (0.005)	1E-04
Height	rs34651	*TNPO1*	No	NA	C	T	Imputed	0.96907	0.042 (0.008)	4E-08
Height	rs354196	*SPTBN1*	No	NA	G	A	Imputed	0.97687	0.006 (0.004)	2E-01
Height	rs3739707	*LPAR1*	No	NA	C	A	Imputed	0.9919	0.029 (0.005)	2E-09
Height	rs3760318	*CENTA2*	No	NA	G	A	Imputed	1	0.051 (0.004)	9E-34
Height	rs3763631	*NPR2/SPAG8*	No	NA	C	G	Imputed	0.99079	0.017 (0.004)	1E-04
Height	rs3782089	*SSSCA1*	No	NA	C	T	Imputed	1	0.027 (0.008)	8E-04
Height	rs3790086	*WWP2*	Yes	SNP not in Hardy-Weinberg equilibrium	C	G	NA	NA	NA	NA
Height	rs3791679	*EFEMP1*	No	NA	A	G	Imputed	1	0.080 (0.005)	9E-60
Height	rs3802758	*PEX16*	No	NA	A	G	Imputed	0.9886	0.009 (0.008)	2E-01
Height	rs3807931	*ITGB8*	No	NA	A	G	Imputed	0.99273	0.031 (0.004)	3E-14
Height	rs3809790	*SSH2*	No	NA	C	T	Imputed	0.99929	0.016	8E-05

									(0.004)	
Height	rs3812040	*DAB2*	No	NA	T	C	Imputed	0.99127	0.018 (0.005)	1E-04
Height	rs3812423	*KCTD9*	No	NA	G	C	Imputed	0.99973	0.013 (0.004)	2E-03
Height	rs3814333	*GLT25D2*	No	NA	T	C	Imputed	1	0.049 (0.004)	2E-28
Height	rs3818416	*EDNRB*	No	NA	C	A	Imputed	0.99548	0.019 (0.005)	7E-05
Height	rs3825199	*SOCS2*	No	NA	G	A	Imputed	1	0.059 (0.005)	2E-32
Height	rs3885668	*KLF11*	No	NA	C	T	Imputed	0.99257	0.023 (0.004)	4E-08
Height	rs3915129	*CTNNB1*	No	NA	G	T	Imputed	0.99872	0.024 (0.004)	3E-09
Height	rs3923086	*AXIN2*	No	NA	C	A	Imputed	0.9707	0.029 (0.004)	6E-12
Height	rs3958122	*SLBP*	No	NA	T	C	Imputed	0.99446	0.025 (0.004)	4E-09
Height	rs39623	*ADAMTS19*	No	NA	A	T	Imputed	0.99499	0.049 (0.008)	2E-10
Height	rs4072910	*ADAMTS10*	No	NA	G	C	Imputed	1	0.033 (0.004)	4E-16
Height	rs42039	*CDK6*	No	NA	T	C	Imputed	0.99565	0.058 (0.005)	4E-34
Height	rs422421	*FGFR4*	No	NA	C	T	Imputed	1	0.044 (0.005)	1E-18
Height	rs4239020	*CCDC57*	No	NA	C	T	Imputed	1	0.017 (0.004)	1E-04
Height	rs425277	*PRKCZ*	No	NA	T	C	Imputed	1	0.018 (0.005)	8E-05
Height	rs429433	*MFHAS1*	No	NA	A	G	Imputed	1	0.045 (0.010)	2E-06
Height	rs4332428	*AKR1C1*	No	NA	A	G	Imputed	0.9991	0.047 (0.006)	2E-13
Height	rs4350272	*ARHGAP21*	No	NA	A	G	Imputed	0.99009	0.012 (0.005)	9E-03
Height	rs4369779	*CABLES1*	No	NA	C	T	Imputed	1	0.075	2E-50

									(0.005)	
Height	rs4425077	*FN1*	No	NA	G	C	Imputed	0.99732	0.008 (0.004)	5E-02
Height	rs4548838	*ADAMTS17*	No	NA	T	C	Imputed	0.99398	0.035 (0.004)	3E-17
Height	rs4601530	*CLIC4*	No	NA	C	T	Imputed	1	0.001 (0.005)	8E-01
Height	rs4605213	*NME1-NME2/NME2*	No	NA	C	G	Imputed	1	0.017 (0.004)	8E-05
Height	rs4624820	*SPRY4*	No	NA	A	G	Imputed	1	0.005 (0.004)	2E-01
Height	rs4640244	*KCNJ12*	No	NA	A	G	Imputed	1	0.028 (0.004)	2E-11
Height	rs4656220	*PRRX1*	No	NA	T	C	Imputed	1	0.017 (0.004)	1E-04
Height	rs4686904	*BCL6*	No	NA	C	T	Imputed	0.99701	0.025 (0.004)	5E-09
Height	rs4725061	*GLCCI1*	No	NA	G	A	Imputed	0.99019	0.021 (0.004)	3E-07
Height	rs4733724	*MLZE*	No	NA	A	G	Imputed	0.99728	0.057 (0.005)	6E-29
Height	rs4735677	*PXMP3*	No	NA	T	A	Imputed	0.99776	0.045 (0.005)	4E-23
Height	rs4785393	*PAPD5*	No	NA	G	A	Imputed	0.98617	0.011 (0.005)	5E-02
Height	rs4802134	*SIPA1L3*	No	NA	A	G	Imputed	0.99656	0.006 (0.005)	2E-01
Height	rs4803468	*BCKDHA*	No	NA	A	G	Imputed	0.99945	0.031 (0.004)	7E-14
Height	rs4812586	*SAMHD1*	No	NA	A	G	Imputed	0.99743	0.035 (0.006)	2E-09
Height	rs4843367	*RAB28*	No	NA	G	A	Imputed	0.99706	0.007 (0.004)	1E-01
Height	rs4868126	*FBXW11*	No	NA	G	T	Imputed	0.93315	0.035 (0.004)	7E-16
Height	rs4875421	*CSMD1*	No	NA	T	A	Imputed	0.99645	0.010 (0.004)	1E-02
Height	rs4883972	*KLF12*	No	NA	C	G	Imputed	0.99117	-0.005	2E-01

									(0.004)	
Height	rs4896582	*GPR126*	No	NA	G	A	Imputed	1	0.058 (0.004)	6E-38
Height	rs4953951	*ZRANB3*	No	NA	C	T	Imputed	0.99645	0.039 (0.003)	1E-31
Height	rs497273	*SPPL3*	No	NA	C	G	Imputed	0.99824	0.019 (0.004)	7E-06
Height	rs4974480	*ANAPC13*	No	NA	T	A	Imputed	0.98669	0.024 (0.004)	8E-08
Height	rs4986172	*ACBD4*	No	NA	C	T	Imputed	1	0.030 (0.004)	7E-12
Height	rs526896	*PITX1*	No	NA	T	G	Imputed	1	0.030 (0.005)	6E-11
Height	rs540652	*NOSTRIN*	No	NA	T	C	Imputed	1	0.024 (0.004)	3E-09
Height	rs552707	*JAZF1*	No	NA	T	C	Imputed	0.99867	0.051 (0.004)	2E-29
Height	rs564914	*FOXD2*	No	NA	T	A	Imputed	1	0.016 (0.004)	1E-04
Height	rs567401	*DDAH1*	Yes	Imputation r2<0.9	T	C	NA	NA	NA	NA
Height	rs568610	*SCARA3*	No	NA	T	C	Imputed	0.98877	0.023 (0.005)	3E-06
Height	rs5742915	*PML*	No	NA	C	T	Imputed	1	0.037 (0.004)	8E-20
Height	rs584828	*IGFBP4*	No	NA	C	T	Imputed	0.99238	0.028 (0.004)	1E-11
Height	rs6061231	*RPS21*	No	NA	C	A	Imputed	0.98656	0.017 (0.005)	2E-04
Height	rs606452	*SERPINH1*	No	NA	A	C	Imputed	1	0.055 (0.006)	1E-20
Height	rs6080830	*BANF2*	No	NA	A	G	Imputed	0.99462	0.016 (0.004)	8E-05
Height	rs632124	*DDX6*	No	NA	A	T	Imputed	0.99825	0.017 (0.004)	4E-05
Height	rs6420435	*MPHOSPH6*	No	NA	A	C	Imputed	0.97978	0.022 (0.005)	5E-06
Height	rs6435143	*NOP5/NOP58*	No	NA	A	C	Imputed	0.98904	0.003 (0.004)	4E-01

Height	rs6439168	*H1FX*	No	NA	G	A	Imputed	0.9904	0.045 (0.005)	1E-19
Height	rs6441170	*SHOX2*	No	NA	C	T	Imputed	0.99934	0.024 (0.004)	1E-08
Height	rs6446315	*CYTL1*	No	NA	G	A	Imputed	0.98569	0.013 (0.006)	2E-02
Height	rs6457374	*HLA-C*	No	NA	C	T	Imputed	1	0.046 (0.004)	8E-25
Height	rs6462432	*KBTBD2*	No	NA	A	G	Imputed	1	0.010 (0.004)	2E-02
Height	rs6485978	*TEAD1*	No	NA	C	T	Imputed	0.99307	0.023 (0.004)	4E-08
Height	rs6540834	*PTPN14*	No	NA	C	T	Imputed	1	0.018 (0.004)	2E-05
Height	rs6561319	*LRCH1*	No	NA	A	C	Imputed	0.99061	0.025 (0.004)	5E-09
Height	rs6584575	*SH3PXD2A*	No	NA	A	G	Imputed	0.98594	0.026 (0.007)	2E-04
Height	rs6600365	*SCMH1*	No	NA	C	T	Imputed	1	0.028 (0.004)	5E-12
Height	rs6658763	*FMO5*	No	NA	C	T	Imputed	1	0.012 (0.008)	1E-01
Height	rs6688100	*VANGL2*	No	NA	T	C	Imputed	1	0.006 (0.004)	1E-01
Height	rs6691924	*ACOT11*	No	NA	T	C	Imputed	1	0.019 (0.006)	3E-03
Height	rs6694089	*DNM3*	No	NA	A	G	Imputed	1	0.035 (0.005)	2E-14
Height	rs6696239	*ZNF678*	No	NA	G	A	Imputed	1	0.043 (0.005)	1E-16
Height	rs6714546	*LTBP1*	No	NA	G	A	Imputed	1	0.036 (0.005)	6E-16
Height	rs6746356	*SP3*	No	NA	A	C	Imputed	0.99407	0.020 (0.005)	2E-05
Height	rs6761041	*SERPINE2*	No	NA	T	C	Imputed	0.98915	0.024 (0.004)	1E-08
Height	rs6794009	*PTPRG*	No	NA	G	A	Imputed	0.9852	0.030 (0.004)	5E-13

Height	rs6813055	*DMP1*	No	NA	A	T	Imputed	0.99764	0.020 (0.004)	7E-07
Height	rs6838153	*EXOSC9*	No	NA	G	A	Imputed	0.98791	0.017 (0.004)	2E-04
Height	rs6879260	*GFPT2*	No	NA	C	T	Imputed	1	0.024 (0.004)	6E-09
Height	rs6894139	*MEF2C*	No	NA	T	G	Imputed	0.99738	0.031 (0.004)	4E-14
Height	rs6902771	*ESR1*	No	NA	T	C	Imputed	1	0.033 (0.004)	8E-16
Height	rs6920372	*PPIL6*	No	NA	G	A	Imputed	0.99527	0.024 (0.004)	6E-09
Height	rs692964	*CEP192*	No	NA	G	A	Imputed	0.99944	0.015 (0.004)	4E-04
Height	rs6949739	*IGFBP3*	No	NA	T	A	Imputed	0.99158	0.037 (0.007)	1E-06
Height	rs6952113	*C7orf58*	No	NA	G	A	Imputed	0.99963	0.019 (0.004)	5E-06
Height	rs6955948	*TMEM176A*	No	NA	T	C	Imputed	0.99692	0.018 (0.005)	7E-05
Height	rs6962887	*CNOT4*	No	NA	T	G	Imputed	0.97958	0.025 (0.004)	3E-08
Height	rs6971575	*SLC25A13*	No	NA	C	G	Imputed	0.97974	0.009 (0.004)	4E-02
Height	rs6974574	*STARD3NL*	No	NA	T	A	Imputed	0.99796	0.026 (0.004)	8E-10
Height	rs6988484	*EFCAB1*	No	NA	C	T	Imputed	0.99665	0.023 (0.005)	1E-06
Height	rs7027110	*ZNF462*	No	NA	A	G	Imputed	1	0.032 (0.005)	8E-11
Height	rs7033487	*PAPPA*	No	NA	T	C	Imputed	0.99616	0.038 (0.005)	2E-13
Height	rs7033940	*UHRF2*	No	NA	G	C	Imputed	0.99	0.011 (0.007)	8E-02
Height	rs7043114	*IPPK*	No	NA	C	T	Imputed	0.99813	0.014 (0.004)	5E-04
Height	rs7069985	*RAB18*	No	NA	G	A	Imputed	0.99489	0.016 (0.005)	1E-03

Height	rs7112925	*RHOD*	No	NA	C	T	Imputed	1	0.034 (0.004)	2E-15
Height	rs7154721	*TRIP11*	No	NA	T	C	Imputed	0.99827	0.026 (0.004)	5E-10
Height	rs7162542	*ADAMTSL3*	No	NA	G	C	Imputed	0.9977	0.048 (0.004)	6E-31
Height	rs7162825	*LACTB*	No	NA	T	C	Imputed	0.99789	0.007 (0.004)	8E-02
Height	rs7177711	*FAM148A*	No	NA	A	G	Imputed	0.99794	0.022 (0.004)	1E-07
Height	rs7181724	*MCTP2*	No	NA	G	A	Imputed	0.96555	0.022 (0.004)	3E-07
Height	rs720390	*IGF2BP2*	No	NA	A	G	Imputed	1	0.034 (0.004)	8E-16
Height	rs724016	*ZBTB38*	No	NA	G	A	Imputed	1	0.085 (0.004)	2E-94
Height	rs7253628	*ZNF536*	No	NA	G	A	Imputed	0.99548	0.029 (0.005)	1E-07
Height	rs7259684	*LOC729747*	No	NA	G	A	Imputed	0.99894	0.047 (0.008)	5E-10
Height	rs7261425	*C20orf26*	No	NA	C	G	Imputed	1	0.015 (0.005)	1E-03
Height	rs7273787	*SMOX*	No	NA	G	A	Imputed	0.99541	0.031 (0.004)	4E-13
Height	rs7284476	*TRIOBP*	No	NA	A	G	Imputed	0.99699	0.017 (0.004)	4E-05
Height	rs7319045	*GPC5*	No	NA	A	G	Imputed	1	0.030 (0.004)	1E-12
Height	rs738288	*SMCR7L*	No	NA	G	A	Imputed	0.98417	0.010 (0.004)	2E-02
Height	rs7466269	*FUBP3*	No	NA	A	G	Imputed	1	0.035 (0.004)	2E-16
Height	rs749234	*ZEB2*	No	NA	A	G	Imputed	0.99884	0.013 (0.004)	4E-03
Height	rs7517682	*COL11A1*	No	NA	G	A	Imputed	1	0.032 (0.004)	9E-15
Height	rs7534365	*SV2A*	Yes	Imputation r2<0.9	C	T	NA	NA	NA	NA
Height	rs7544462	*C1orf149*	No	NA	A	C	Imputed	1	0.035	2E-06

									(0.007)	
Height	rs7551732	*PKN2*	No	NA	A	T	Imputed	1	0.029 (0.004)	2E-12
Height	rs7567288	*NAP5*	No	NA	C	T	Imputed	1	0.017 (0.005)	9E-04
Height	rs7567851	*PDE11A*	No	NA	C	G	Imputed	1	0.027 (0.008)	4E-04
Height	rs7568069	*ZNF638*	No	NA	G	A	Imputed	0.99949	0.031 (0.004)	2E-13
Height	rs757081	*NUCB2*	No	NA	G	C	Imputed	1	0.019 (0.004)	1E-05
Height	rs761391	*TBX18*	No	NA	C	T	Imputed	0.99562	0.015 (0.004)	3E-04
Height	rs763318	*RAB28*	No	NA	G	A	Imputed	0.98628	0.031 (0.004)	2E-14
Height	rs7652177	*FNDC3B*	No	NA	G	C	Imputed	1	0.031 (0.004)	2E-14
Height	rs7659107	*CAMK2D*	No	NA	G	A	Imputed	0.99028	0.004 (0.005)	4E-01
Height	rs7692995	*LCORL*	Yes	SNP not in Hardy-Weinberg equilibrium	T	C	NA	NA	NA	NA
Height	rs7701414	*PDLIM4*	No	NA	G	A	Imputed	0.99613	0.043 (0.004)	9E-26
Height	rs7716219	*SLC38A9*	No	NA	T	C	Imputed	0.9953	0.035 (0.004)	6E-15
Height	rs7727731	*ADAMTS6*	No	NA	T	C	Imputed	0.99557	0.020 (0.006)	1E-03
Height	rs7733195	*FAM44B*	No	NA	G	A	Imputed	0.99698	0.022 (0.004)	4E-07
Height	rs7740107	*L3MBTL3*	No	NA	T	A	Imputed	0.99629	0.062 (0.005)	2E-40
Height	rs780094	*GCKR*	No	NA	C	T	Imputed	1	0.022 (0.004)	2E-07
Height	rs7834383	*DLC1*	No	NA	T	G	Imputed	0.98385	0.011 (0.004)	1E-02
Height	rs7849585	*QSOX2*	No	NA	T	G	Imputed	0.99758	0.031 (0.004)	2E-12
Height	rs7853235	*RMI1*	No	NA	T	C	Imputed	0.98874	0.030	7E-09

									(0.005)	
Height	rs7899004	*SUFU*	No	NA	T	C	Imputed	0.99647	0.035 (0.004)	1E-17
Height	rs7971536	*CCDC53*	No	NA	T	A	Imputed	0.99239	0.028 (0.004)	5E-12
Height	rs7980687	*SBNO1*	No	NA	A	G	Imputed	1	0.043 (0.005)	1E-17
Height	rs798497	*GNA12*	No	NA	A	G	Imputed	1	0.062 (0.004)	1E-43
Height	rs7985356	*CDC16*	No	NA	T	A	Imputed	0.99639	0.026 (0.005)	1E-07
Height	rs8006657	*SAMD4A*	No	NA	G	A	Imputed	0.97777	0.014 (0.004)	1E-03
Height	rs8017130	*HOMEZ*	No	NA	G	A	Imputed	0.96175	0.017 (0.004)	1E-04
Height	rs8052560	*C16orf84*	No	NA	A	C	Imputed	1	0.031 (0.005)	2E-10
Height	rs8058684	*RBL2*	No	NA	A	G	Imputed	0.99637	0.031 (0.004)	3E-12
Height	rs806794	*HIST1H2BF*	No	NA	A	G	Imputed	1	0.063 (0.005)	4E-43
Height	rs8097893	*GALR1*	No	NA	A	G	Imputed	0.99143	0.037 (0.010)	3E-04
Height	rs8102380	*ILF3*	No	NA	G	A	Imputed	0.99499	0.024 (0.004)	3E-08
Height	rs8103068	*BST2*	No	NA	T	C	Imputed	0.99332	0.024 (0.006)	1E-04
Height	rs8103992	*PBX4*	No	NA	A	C	Imputed	0.98893	0.032 (0.005)	9E-10
Height	rs817300	*PTCH1*	No	NA	G	A	Imputed	1	0.082 (0.008)	2E-26
Height	rs8180991	*TRIB1*	No	NA	C	G	Imputed	0.99501	0.026 (0.005)	3E-08
Height	rs820848	*HEXB*	No	NA	G	A	Imputed	0.98738	0.012 (0.005)	1E-02
Height	rs822531	*EZH2*	No	NA	T	C	Imputed	0.97338	0.053 (0.005)	6E-25
Height	rs833152	*PDE1A*	No	NA	C	A	Imputed	0.98533	0.020	2E-06

									(0.004)	
Height	rs862034	*LTBP2*	No	NA	G	A	Imputed	1	0.027 (0.004)	3E-10
Height	rs870183	*VPS53*	No	NA	G	A	Imputed	0.99432	0.001 (0.004)	8E-01
Height	rs8756	*HMGA2*	No	NA	C	A	Imputed	1	0.056 (0.004)	4E-42
Height	rs888403	*SMCHD1*	No	NA	G	A	Imputed	0.99488	0.011 (0.004)	8E-03
Height	rs891088	*INSR*	No	NA	G	A	Imputed	1	0.027 (0.005)	9E-09
Height	rs897080	*C2orf34*	No	NA	C	T	Imputed	0.99077	0.026 (0.005)	9E-08
Height	rs915506	*CCNJ*	Yes	SNP not in Hardy-Weinberg equilibrium	G	A	NA	NA	NA	NA
Height	rs9217	*ZBTB4*	No	NA	C	T	Imputed	1	0.043 (0.004)	2E-24
Height	rs9291926	*PIK3R1*	No	NA	T	G	Imputed	0.99821	0.021 (0.004)	3E-07
Height	rs9292468	*C5orf23*	No	NA	T	C	Imputed	0.99566	0.034 (0.004)	3E-16
Height	rs929637	*TMEM106B*	No	NA	G	T	Imputed	0.99652	0.014 (0.005)	3E-03
Height	rs9309101	*THADA*	No	NA	G	A	Imputed	0.98804	0.014 (0.004)	8E-04
Height	rs932445	*GMDS*	No	NA	T	C	Imputed	0.99851	0.016 (0.004)	9E-05
Height	rs936339	*PCOLCE2*	No	NA	T	C	Imputed	0.98118	0.016 (0.005)	2E-03
Height	rs9392918	*BMP6*	No	NA	C	T	Imputed	0.99254	0.050 (0.004)	4E-34
Height	rs9395264	*CD2AP*	No	NA	G	T	Imputed	0.99488	0.026 (0.004)	5E-09
Height	rs9404952	*HLA-G*	No	NA	A	G	Imputed	0.99737	0.003 (0.004)	5E-01
Height	rs9428104	*SPAG17*	No	NA	G	A	Imputed	1	0.049 (0.005)	1E-25
Height	rs9434723	*H6PD*	No	NA	A	G	Imputed	1	0.024	6E-05

									(0.006)	
Height	rs955748	*WWC2*	No	NA	G	A	Imputed	1	0.019 (0.005)	7E-05
Height	rs9650315	*CHCHD7*	No	NA	G	T	Imputed	1	0.066 (0.006)	3E-27
Height	rs975210	*TLE3*	No	NA	A	G	Imputed	0.98941	0.035 (0.005)	5E-11
Height	rs9766	*EZH1*	No	NA	A	G	Imputed	0.99396	0.019 (0.004)	5E-06
Height	rs9816693	*VILL*	No	NA	C	G	Imputed	1	0.023 (0.005)	1E-05
Height	rs9825951	*COL8A1*	No	NA	T	A	Imputed	0.98378	0.019 (0.004)	6E-06
Height	rs9835332	*C3orf63*	No	NA	G	C	Imputed	1	0.024 (0.004)	5E-09
Height	rs9841435	*CCDC50*	No	NA	G	A	Imputed	0.99917	0.011 (0.004)	1E-02
Height	rs9858528	*KLHL24*	No	NA	A	G	Imputed	0.99428	0.000 (0.005)	9E-01
Height	rs9880211	*STAG1*	No	NA	G	A	Imputed	0.99892	0.024 (0.005)	3E-07
Height	rs989393	*COL15A1*	No	NA	T	C	Imputed	0.99371	0.019 (0.005)	2E-05
Height	rs991946	*T*	No	NA	C	T	Imputed	0.99381	0.022 (0.004)	4E-08
Height	rs991967	*TGFB2*	No	NA	C	A	Imputed	1	0.049 (0.005)	4E-27
Height	rs9967417	*DYM*	No	NA	G	C	Imputed	1	0.040 (0.004)	3E-22
Height	rs9977276	*COL6A1*	No	NA	G	T	Imputed	0.99096	0.026 (0.005)	2E-07
Height	rs9993613	*ADAMTS3*	No	NA	T	G	Imputed	0.99351	0.040 (0.004)	7E-22

Supplementary Table D: Observational and genetic associations for A) height and B) body mass index (BMI) with job class and annual household income using ordinal regression models.

A

			**Observational associations**		**Genetic associations**	
**Socio economic status measure**	**Subcategories**	**N**	**Odds ratio (95%CI) for higher socioeconomic status SD increase in height**	**P**	**Odds ratio (95%CI) for higher socioeconomic status per SD increase in height**	**P**
Job class	All	76404	1.22 (1.20 to 1.23)	<1E-15	1.08 (1.04 to 1.11)	7E-5
	Male only	37608	1.22 (1.20 to 1.24)	<1E-15	1.09 (1.04 to 1.15)	0.0004
	Female only	38796	1.21 (1.19 to 1.23)	<1E-15	1.04 (0.99 to 1.10)	0.08
Annual household income	All	103327	1.26 (1.24 to 1.27)	<1E-15	1.09 (1.06 to 1.13)	2E-8
	Male only	50862	1.30 (1.28 to 1.32)	<1E-15	1.14 (1.10 to 1.19)	5E-10
	Female only	52465	1.21 (1.19 to 1.23)	<1E-15	1.04 (0.99 to 1.09)	0.09

B

			**Observational associations**		**Genetic associations**	
**Socio economic status measure**	**Subcategories**	**N**	**Odds ratio (95%CI) for higher socioeconomic status per SD higher BMI**	**P**	**Odds ratio (95%CI) for higher socioeconomic status per SD higher BMI**	**P**
Job class	All	76404	0.94 (0.93 to 0.96)	<1E-15	0.89 (0.81 to 0.99)	0.029
	Male only	37608	0.99 (0.97 to 1.01)	0.16	0.90 (0.77 to 1.06)	0.22
	Female only	38796	0.91 (0.90 to 0.93)	<1E-15	0.89 (0.78 to 1.01)	0.07
Annual household income	All	103327	0.90 (0.89 to 0.91)	<1E-15	0.89 (0.81 to 0.97)	0.01
	Male only	50862	0.98 (0.96 to 0.99)	0.005	1.08 (0.94 to 1.24)	0.29

Female only	52465	0.86 (0.84 to 0.87)	<1E-15	0.76 (0.68 to 0.86)	5E-6

Supplementary table E: Genetic associations between BMI and income in women stratified on A) employment status and marital status or B) health status

Employment Status	Marital status	N	Beta (95%CI) representing a SD change in income per SD change in BMI	P
Working	All	33,939	-0.15 (-0.23 to -0.07)	4E-4
Working	Live with husband/partner	23,575	-0.12 (-0.22 to -0.03)	0.01
Working	Do not live with husband/partner	9,563	-0.10 (-0.22 to 0.01)	0.08
Non-working	All	28,914	-0.11 (-0.21 to -0.01)	0.03
Non-working	Live with husband/partner	20,305	-0.12 (-0.25 to 0.01)	0.07
Non-working	Do not live with husband/partner	6,981	0.01 (-0.10 to 0.13)	0.82

Health status	N	Beta (95%CI) representing a SD change in income per SD change in BMI	P
No non-cancer or cancer disease reported	12,127	-0.23 (-0.39, -0.08)	0.004

Note marital status classified as living with husband or partner versus those not living with a husband/partner

Supplementary table F: Associations between taller stature and five measures of socio-economic comparing standard instrumental variable analysis and the Egger method

Socio economic status measure	Subcategories	N	Genetic^ Change in socioeconomic status (95%CI) per SD taller stature	P	Genetic – Egger^^ Change in socioeconomic status (95%CI) per SD taller stature	P
Age completed full time education	All	82543	0.03 (0.01 to 0.05)	0.01	0.07 (0.03 to 0.11)	0.0004
	Male only	38342	0.04 (0.01 to 0.07)	0.009	0.08 (0.02 to 0.14)	0.004
	Female only	44201	0.01 (-0.02 to 0.04)	0.40	0.05 (0.01 to 0.09)	0.018
Degree education	All	118565	1.02 (0.99 to 1.05)	0.22	1.06 (0.99 to 1.13)	0.09
	Male only	56111	1.04 (1.00 to 1.09)	0.08	1.10 (1.01 to 1.19)	0.026
	Female only	62454	1.00 (0.95 to 1.05)	0.97	1.09 (1.00 to 1.19)	0.06
Job class (skilled/unskilled)	All	76404	1.12 (1.07 to 1.18)	6E-7	1.18 (1.08 to 1.29)	0.0002
	Male only	37608	1.13 (1.07 to 1.21)	2E-5	1.23 (1.10 to 1.37)	0.0004
	Female only	38796	1.14 (1.05 to 1.24)	0.003	1.21 (1.08 to 1.36)	0.002
Annual household income	All	103327	0.05 (0.03 to 0.07)	4E-8	0.05 (0.02 to 0.08)	0.0009
	Male only	50862	0.07 (0.05 to 0.10)	1E-9	0.08 (0.04 to 0.12)	0.0002
	Female only	52465	0.02 (0.00 to 0.05)	0.09	0.05 (0.00 to 0.10)	0.09
Townsend deprivation index	All	119519	0.00 (-0.02 to 0.01)	0.71	-0.03 (-0.06 to 0.00)	0.038
	Male only	56582	-0.02 (-0.05 to 0.00)	0.05	-0.08 (-0.12 to -0.04)	0.0004
	Female only	62937	0.02 (-0.01 to 0.04)	0.19	-0.005 (-0.05 to 0.04)	0.8

^Utilises instrumental variable analysis via the ivreg2 command in STATA for continuous variables and the 2-step procedure for binary outcomes using the height Genetic Risk Score. The F-stat when considering all individuals is ≥10898 for each socioeconomic status measure, in males only the F-stat is ≥5308 for each socioeconomic status measure and in females only the F-stat is ≥5615 for each socioeconomic status measure.

^^An alternative genetic approach detailed in Bowden et al., 2015

For age completed full time education, annual household income and Townsend deprivation index the changes reported are standard deviation. For degree and job class odds ratios are presented, representing odds of higher socioeconomic status per SD higher height.

Supplementary table G: Sensitivity analysis with the Egger Method to further investigate associations between higher BMI and two measures of socio-economic status

			Genetic^^		Genetic Egger^^^	
**Socio economic status measure**	**Subcategories**	**N**	**Change in socioeconomic status (95%CI) per SD higher BMI**	**P**	**Change in socioeconomic status (95%CI) per SD higher BMI**	**P**
Age completed full time education	All	82543	-0.01 (-0.07 to 0.04)	0.63	0.04 (-0.05 to 0.13)	0.44
	Male only	38342	0.00 (-0.09 to 0.09)	0.98	0.12 (-0.01 to 0.25)	0.08
	Female only	44201	-0.02 (-0.09 to 0.05)	0.56	-0.03 (-0.14 to 0.08)	0.38
Degree education	All	118565	0.94 (0.85 to 1.03)	0.18	1.21 (1.01 to 1.45)	0.038
	Male only	56111	0.94 (0.81 to 1.09)	0.43	1.58 (1.23 to 2.02)	0.0006
	Female only	62454	0.93 (0.82 to 1.06)	0.28	0.97 (0.78 to 1.21)	0.82
Job class (skilled/unskilled)	All	76404	0.90 (0.79 to 1.02)	0.10	0.99 (0.78 to 1.26)	0.93
	Male only	37608	0.88 (0.73 to 1.08)	0.22	1.07 (0.80 to 1.44)	0.65
	Female only	38796	0.91 (0.76 to 1.08)	0.29	0.76 (0.56 to 1.02)	0.08
Annual household income	All	103327	-0.05 (-0.10 to -0.00)	0.041	-0.03 (-0.11 to 0.05)	0.58
	Male only	50862	0.06 (-0.02 to 0.14)	0.15	0.16 (0.04 to 0.29)	0.012
	Female only	52465	-0.14 (-0.20 to -0.08)	1E-5	-0.17 (-0.25 to -0.05)	0.004
Townsend deprivation index	All	119519	0.05 (0.01 to 0.10)	0.024	-0.00 (-0.08 to 0.08)	0.96
	Male only	56582	-0.01 (-0.08 to 0.06)	0.78	-0.12 (-0.23. -0.01)	0.032
	Female only	62937	0.10 (0.04 to 0.16)	0.001	0.10 (-0.01 to 0.21)	0.08

^Utilises instrumental variable analysis, via the ivreg2 command in STATA for continuous variables and the 2-step approach for binary outcomes, using the BMI Genetic Risk Score. The F-stat for all individuals is ≥1257 for each socioeconomic status measure, in males only the F-stat is ≥591 for each socioeconomic status measure and in females only the F-stat is ≥666 for each socioeconomic status measure.

^^An alternative genetic approach detailed in Bowden et al., 2015

For age completed full time education and Townsend deprivation index the changes reported are standard deviation.

Supplementary table H: Comparison of the A) height and B) BMI associations with the different socioeconomic status measures using the standard method and the linear mixed models that correct for close and distant relatedness, as implemented in BOLT LMM (*2*).

A

**Socioeconomic status measure**	**Method used^**	**Beta (95%CI) for a change in socioeconomic status measure per SD change in height using standard method^^**	**P**	**Beta (95%CI) for a change in socioeconomic status measure per SD change in height using BOLT LMM method^^^**	**P**
Age completed full time education	Genetic	0.03 (0.01 to 0.05)	0.0005	0.04 (0.02 to 0.06)	1E-05
Age completed full time education	Genetic-Egger	0.07 (0.03 to 0.10)	0.0004	0.05 (0.02 to 0.09)	0.005
Job class	Genetic	0.04 (0.02 to 0.06)	2E-05	0.05 (0.03 to 0.07)	1.E-06
Job class	Genetic-Egger	0.06 (0.02 to 0.09)	0.004	0.05 (0.01 to 0.09)	0.016
Income	Genetic	0.06 (0.04 to 0.07)	2E-12	0.05 (0.03 to 0.07)	2E-09
Income	Genetic-Egger	0.05 (0.02 to 0.09)	0.0009	0.04 (0.01 to 0.08)	0.007
Townsend Deprivation index	Genetic	-0.01 (-0.02 to 0.01)	0.14	-0.02 (-0.04 to 0.00)	0.015
Townsend Deprivation index	Genetic-Egger	-0.03 (-0.06 to 0.00)	0.038	-0.04 (-0.07 to -0.01)	0.018

B

**Socioeconomic status measure**	**Method used**	**Beta (95%CI) for a change in socioeconomic status measure per SD change in BMI using standard method^**	**P**	**Beta (95%CI) for a change in socioeconomic status measure per SD change in BMI using BOLT LMM method^^**	**P**

Age completed full time education	Genetic-IV	-0.02 (-0.07 to 0.03)	0.48	-0.04 (-0.08 to 0.01)	0.14
Job class	Genetic-IV	-0.07 (-0.12 to -0.02)	0.011	-0.08 (-0.13 to -0.02)	0.007
Job class	Genetic-Egger	-0.04 (-0.15 to 0.07)	0.44	-0.02 (-0.13 to 0.08)	0.66
Income	Genetic	-0.06 (-0.10 to -0.01)	0.017	-0.07 (-0.12 to -0.02)	0.003
Income	Genetic-Egger	-0.03 (-0.11 to 0.06)	0.58	-0.01 (-0.09 to 0.07)	0.76
Townsend Deprivation index	Genetic	0.05 (0.01 to 0.10)	0.015	0.05 (0.01 to 0.09)	0.015
Townsend Deprivation index	Genetic-Egger	0.00 (-0.08 to 0.08)	0.96	-0.02 (-0.09 to 0.05)	0.63

^ Genetic represents the standard instrumental variables approach and the results from the Egger method were presented as a sensitivity analysis when the standard instrumental variable method was P<0.05 in either the standard method or BOLT-LMM.

^^ Standard method accounts for population stratification by taking the residuals of the exposure and outcome variables for standard linear regression using 9 covariates (age, sex, assessment centre location, 5 (within UK) ancestry principal components and microarray used to measure genotypes. These residualised variables where then inverse normalised.

^^^ The BOLT-LMM approach uses a linear mixed models methodology and corrects for all levels of inter-individual correlation of genotypes due to relatedness, from close relatives to cryptic relatedness caused by population stratification. We inverse normalised the socioeconomic status measures, then took the residuals using 3 covariates (age, sex, assessment centre location) and then inverse normalised again. Dichotomous traits were also tested using BOLT-LMM and consistent p-values were observed, but were not reported here due to the differences in the BOLT-LMM model for handling dichotomous traits.

Supplementary Figures

Supplementary figure A: Forest plots of the observational and genetic associations between a 1SD higher height and socioeconomic status: I) Age completed full time education; II) degree education; III) Job class; IV) Income; V) Townsend deprivation index. The plots display the observational association (Observational) and the genetic association using instrumental variable analysis with the genetic risk score (Genetic-instrumental variables). Higher and lower socioeconomic status are marked on the forest plots as higher SES and lower SES.

Supplementary figure B: Forest plots of the observational and genetic associations between a 1SD higher BMI and socioeconomic status: I) Age completed full time education; II) degree education; III) Job class; IV) Income; V) Townsend deprivation index. The plots display the observational association (Observational) and the genetic association using instrumental variable analysis with the genetic risk score (Genetic-instrumental variables). Higher and lower socioeconomic status are marked on the forest plots as higher SES and lower SES.

Supplementary figure A:

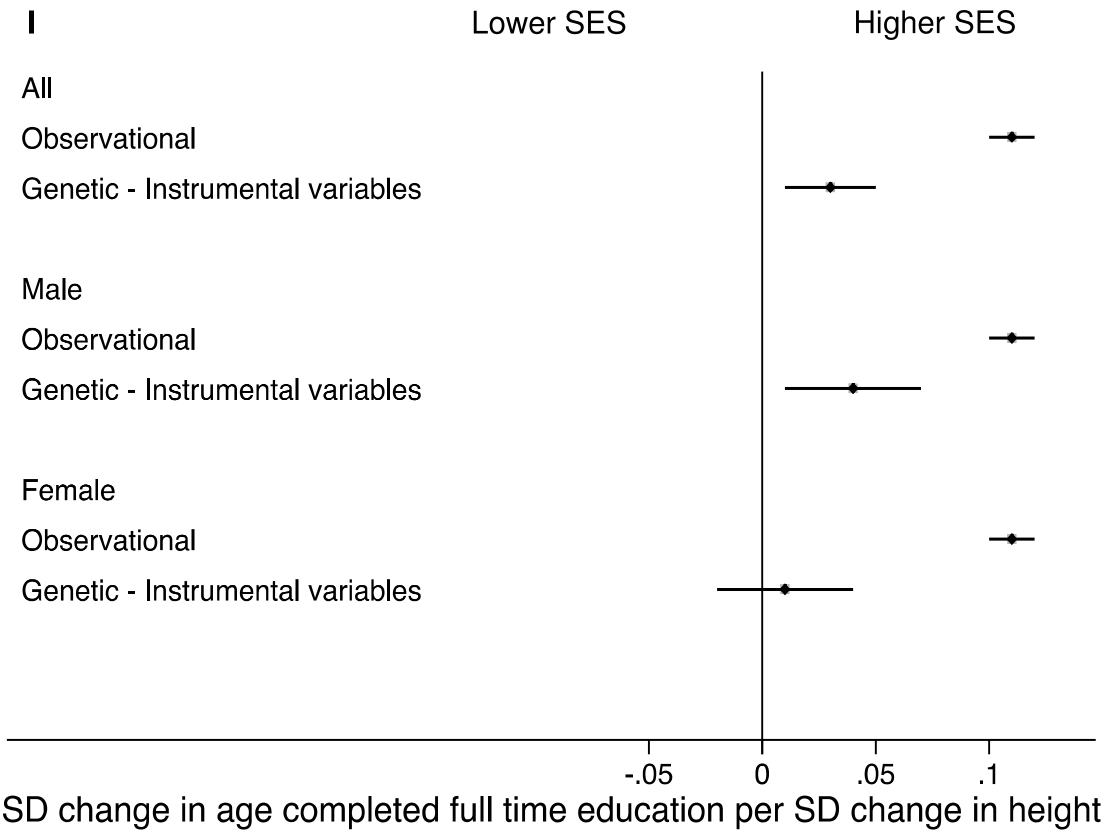

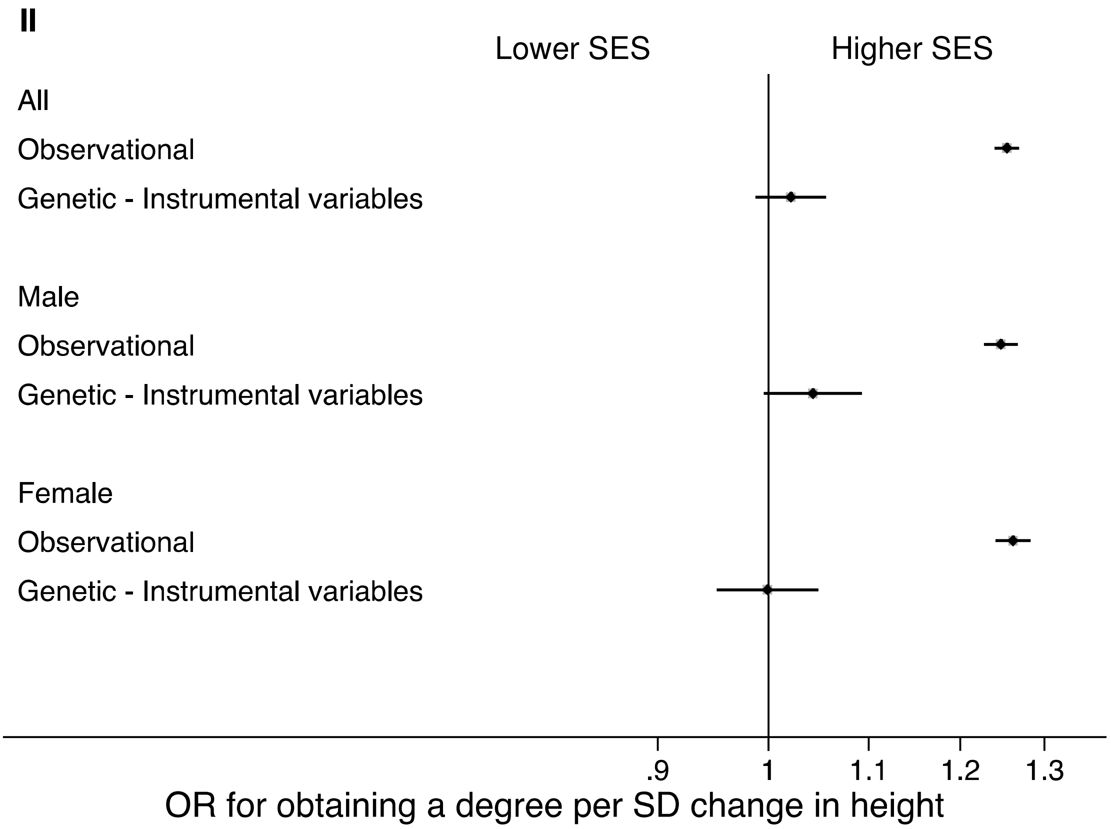

II
Lower SES
Higher SES
All
Observational
Genetic - Instrumental variables
Male
Observational
Genetic - Instrumental variables
Female
Observational
Genetic - Instrumental variables
.9
1
1.1
1.2
1.3
OR for obtaining a degree per SD change in height

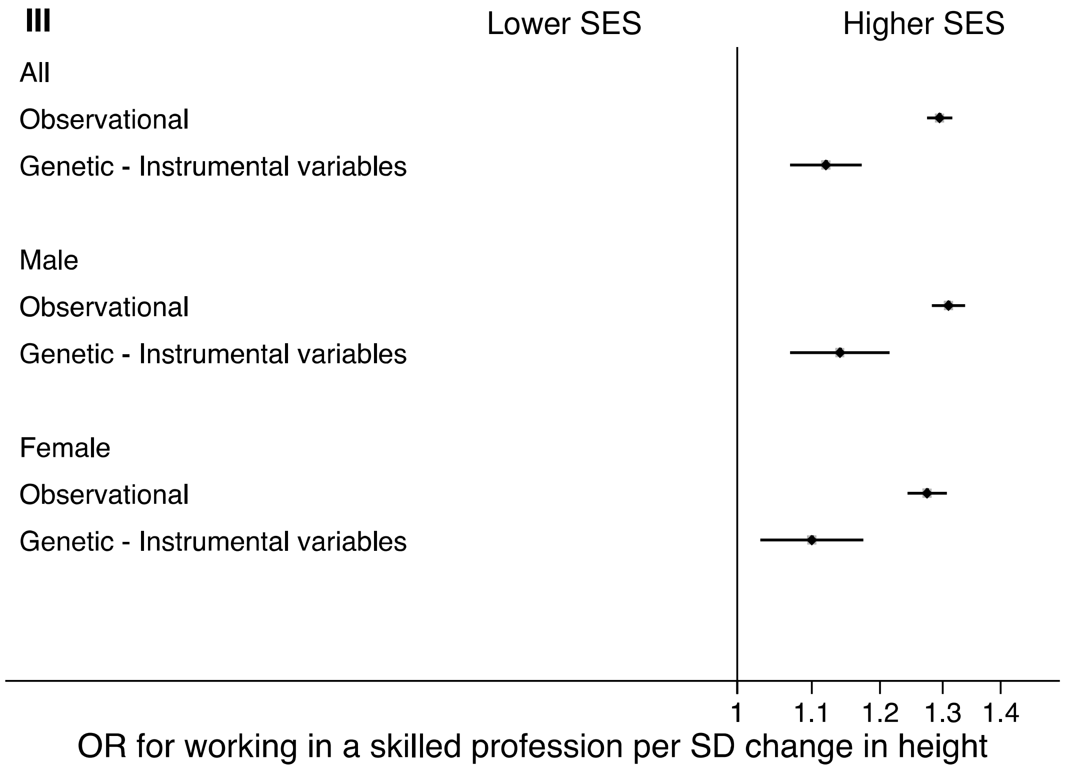
III
Lower SES
Higher SES
All
Observational
Genetic - Instrumental variables
Male
Observational
Genetic - Instrumental variables
Female
Observational
Genetic - Instrumental variables
1
1.1
1.2
1.3
1.4
OR for working in a skilled profession per SD change in height

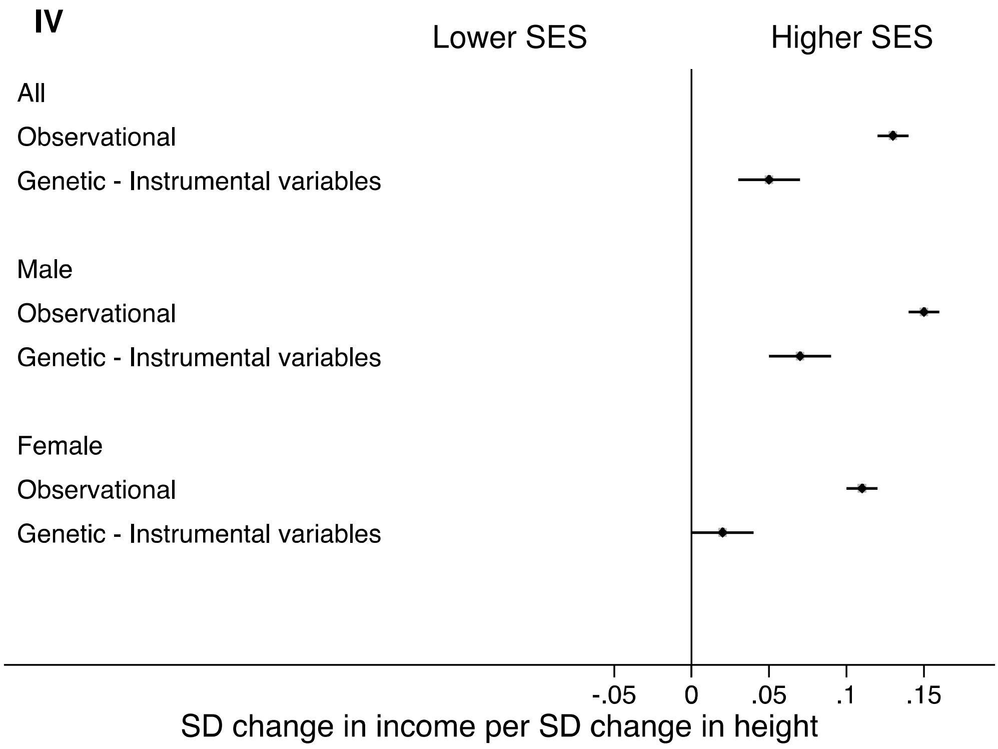
IV
Lower SES
Higher SES
All
Observational
Genetic - Instrumental variables
Male
Observational
Genetic - Instrumental variables
Female
Observational
Genetic - Instrumental variables
-.05
0
.05
.1
.15
SD change in income per SD change in height

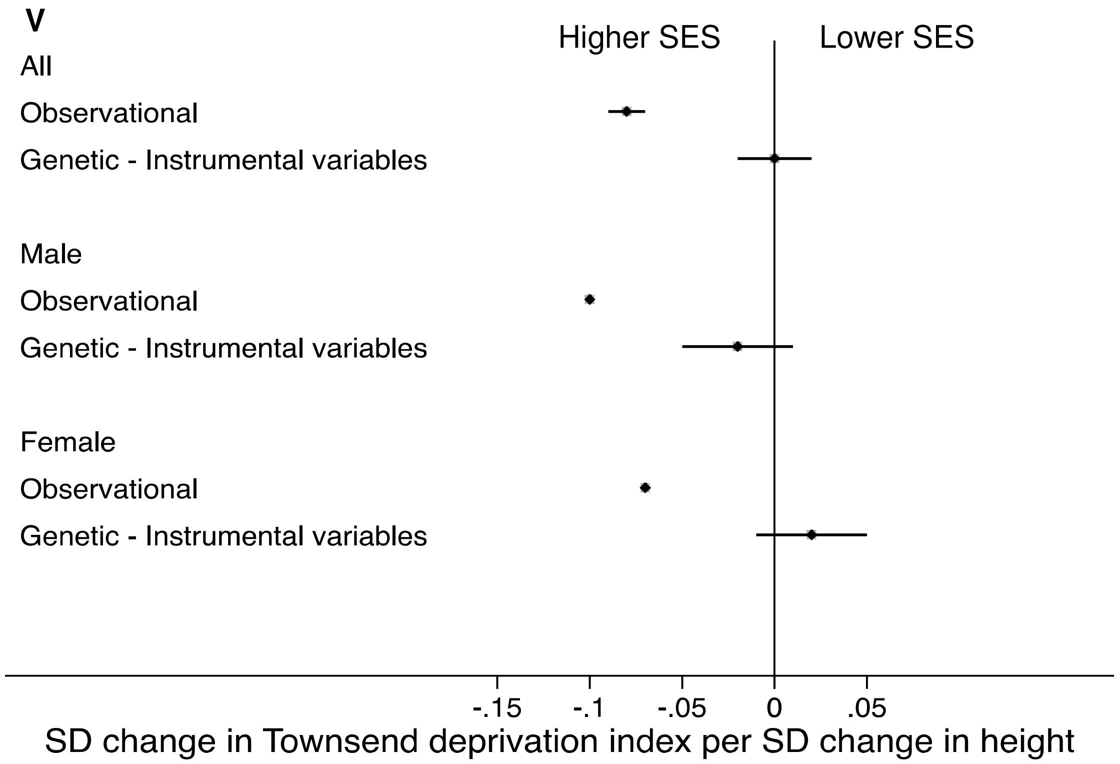
V
Higher SES
Lower SES
All
Observational
Genetic - Instrumental variables
Male
Observational
Genetic - Instrumental variables
Female
Observational
Genetic - Instrumental variables
-.15
-.1
-.05
0
.05
SD change in Townsend deprivation index per SD change in height

Supplementary figure B:

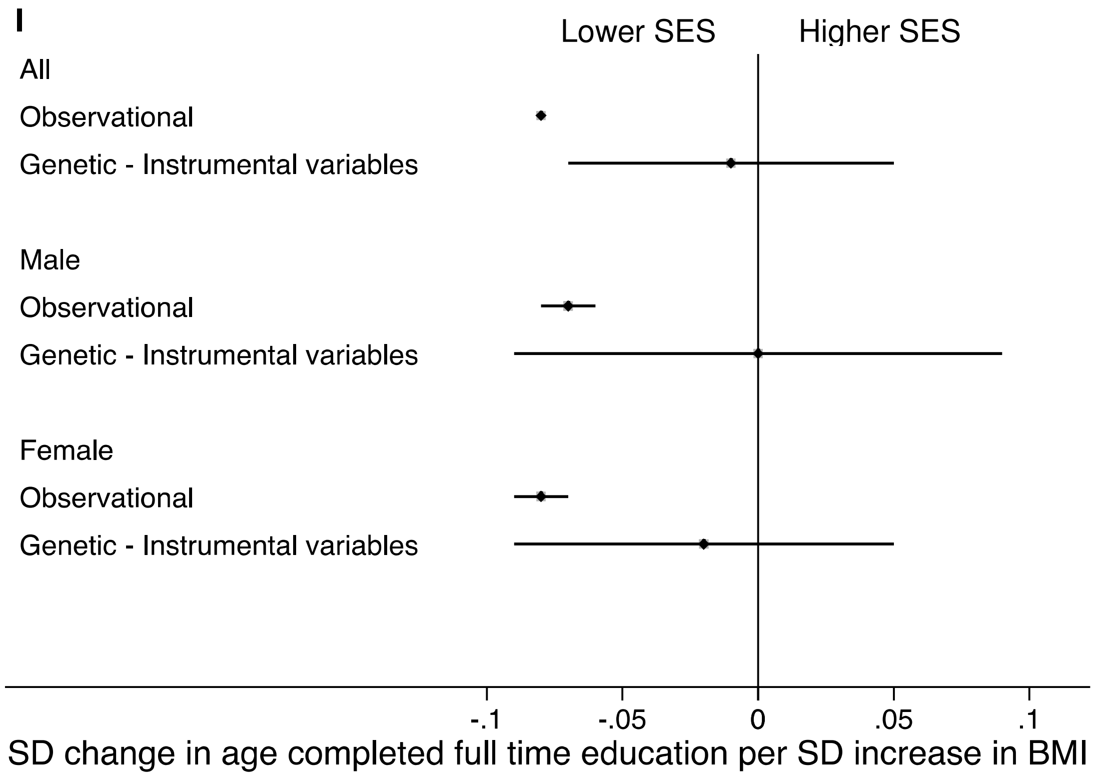

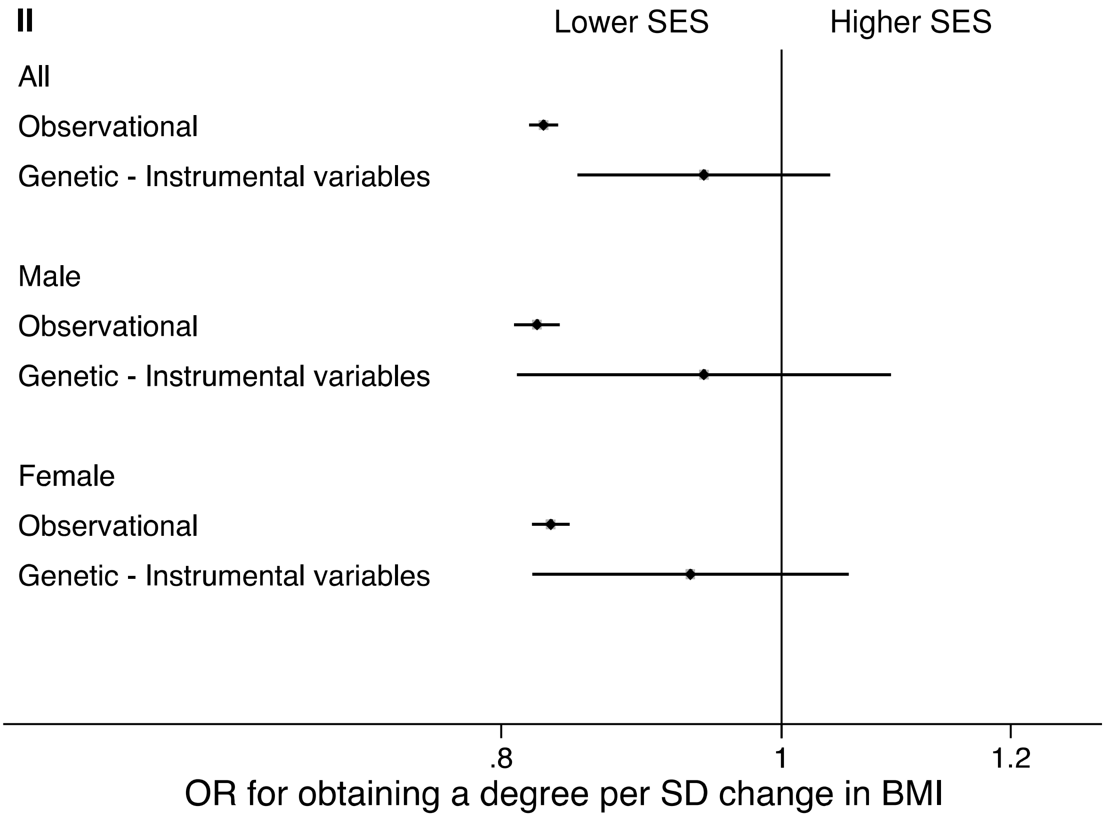
II
Lower SES
Higher SES
All
Observational
Genetic - Instrumental variables
Male
Observational
Genetic - Instrumental variables
Female
Observational
Genetic - Instrumental variables
.8
1
1.2
OR for obtaining a degree per SD change in BMI

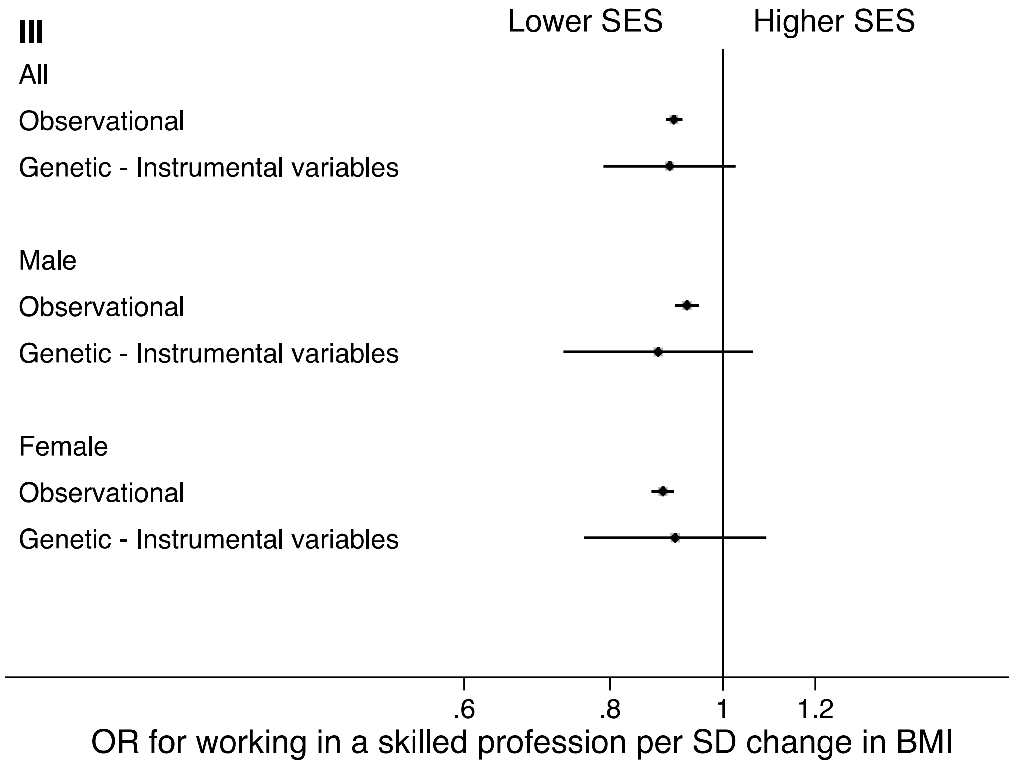
III
Lower SES
Higher SES
All
Observational
Genetic - Instrumental variables
Male
Observational
Genetic - Instrumental variables
Female
Observational
Genetic - Instrumental variables
.6
.8
1
1.2
OR for working in a skilled profession per SD change in BMI

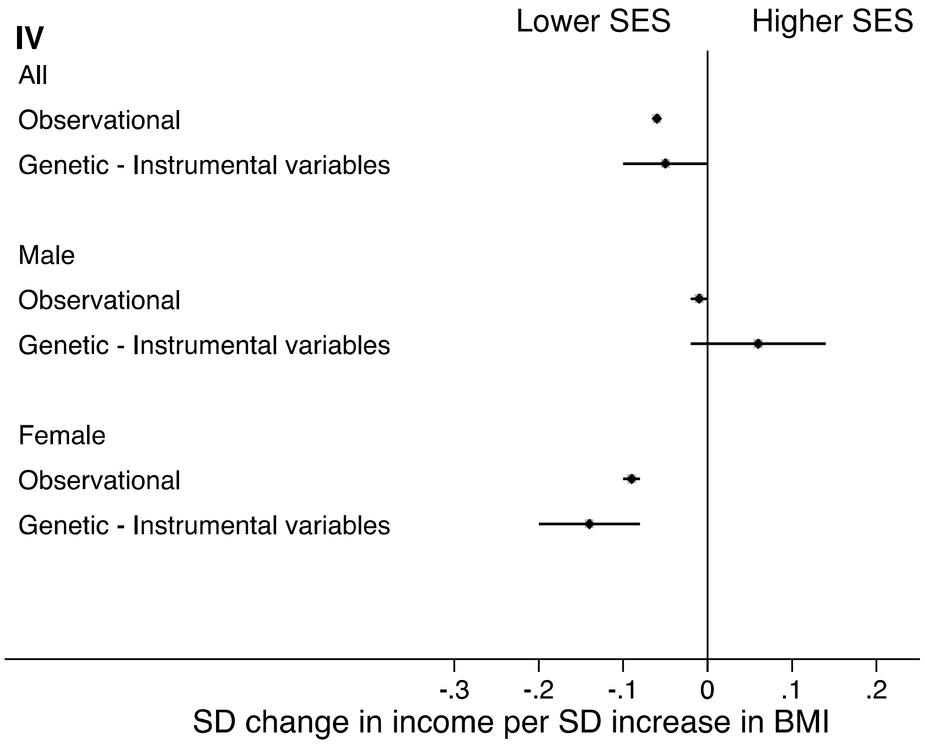
IV
Lower SES
Higher SES
All
Observational
Genetic - Instrumental variables
Male
Observational
Genetic - Instrumental variables
Female
Observational
Genetic - Instrumental variables
-.3 -.2 -.1 0 .1 .2
SD change in income per SD increase in BMI

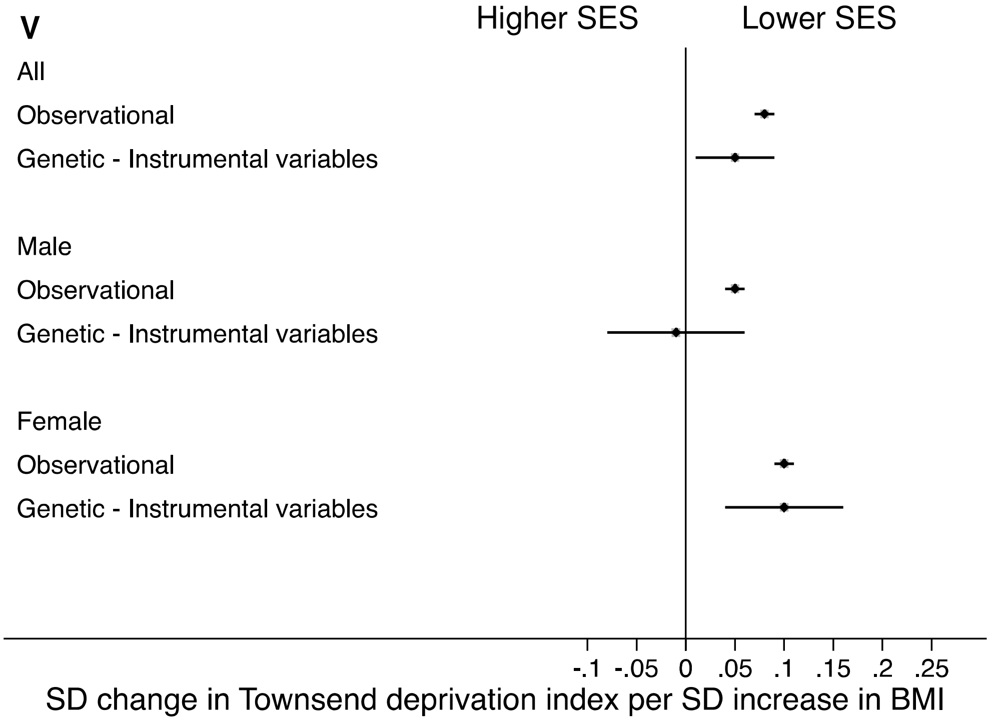
V
Higher SES
Lower SES
All
Observational
Genetic - Instrumental variables
Male
Observational
Genetic - Instrumental variables
Female
Observational
Genetic - Instrumental variables
-.1 -.05 0 .05 .1 .15 .2 .25
SD change in Townsend deprivation index per SD increase in BMI

Supplementary references

1. G. Abraham, M. Inouye, Fast principal component analysis of large-scale genome-wide data. *PLoS One* **9**, e93766 (2014).
2. P. R. Loh *et al.*, Efficient Bayesian mixed-model analysis increases association power in large cohorts. *Nature genetics* **47**, 284-290 (2015).
